# Exploring the dermotoxicity of the mycotoxin deoxynivalenol: combined morphologic and proteomic profiling of human epidermal cells reveals alteration of lipid biosynthesis machinery and membrane structural integrity relevant for skin barrier function

**DOI:** 10.1007/s00204-021-03042-y

**Published:** 2021-04-23

**Authors:** Giorgia Del Favero, Lukas Janker, Benjamin Neuditschko, Julia Hohenbichler, Endre Kiss, Lydia Woelflingseder, Christopher Gerner, Doris Marko

**Affiliations:** 1 Department of Food Chemistry and Toxicology, Faculty of Chemistry, University of Vienna, Währingerstr. 38-40, 1090 Vienna, Austria; 2 Core Facility Multimodal Imaging, Faculty of Chemistry, University of Vienna, Währingerstr. 38-40, 1090 Vienna, Austria; 3 Department of Analytical Chemistry, Faculty of Chemistry, University of Vienna, Währingerstr. 38-40, 1090 Vienna, Austria; 4 Joint Metabolome Facility, Faculty of Chemistry, University of Vienna, Währingerstr. 38-40, 1090 Vienna, Austria; 5 Department of Inorganic Chemistry, Faculty of Chemistry, University of Vienna, Währingerstr. 42, 1090 Vienna, Austria

**Keywords:** Deoxynivalenol dermotoxicity, Skin barrier, Unfolded protein response mitochondrial, Klf4 transcription factor

## Abstract

Deoxynivalenol (vomitoxin, DON) is a secondary metabolite produced by *Fusarium *spp*.* fungi and it is one of the most prevalent mycotoxins worldwide. Crop infestation results not only in food and feed contamination, but also in direct dermal exposure, especially during harvest and food processing. To investigate the potential dermotoxicity of DON, epidermoid squamous cell carcinoma cells A431 were compared to primary human neonatal keratinocytes (HEKn) cells via proteome/phosphoproteome profiling. In A431 cells, 10 µM DON significantly down-regulated ribosomal proteins, as well as mitochondrial respiratory chain elements (OXPHOS regulation) and transport proteins (TOMM22; TOMM40; TOMM70A). Mitochondrial impairment was reflected in altered metabolic competence, apparently combined with interference of the lipid biosynthesis machinery. Functional effects on the cell membrane were confirmed by live cell imaging and membrane fluidity assays (0.1–10 µM DON). Moreover, a common denominator for both A431 and HEKn cells was a significant downregulation of the squalene synthase (FDFT1). In sum, proteome alterations could be traced back to the transcription factor Klf4, a crucial regulator of skin barrier function. Overall, these results describe decisive molecular events sustaining the capability of DON to impair skin barrier function. Proteome data generated in the study are fully accessible via ProteomeXchange with the accession numbers PXD011474 and PXD013613.

**Supplementary Information:**

The online version contains supplementary material available at 10.1007/s00204-021-03042-y.

## Introduction

Skin structural integrity is essential for the maintenance of barrier function. Similarly, at cellular level, the plasma membrane represents the interface with the extracellular environment and, as such, its structure and stability are essential for cellular homeostasis. From a toxicological perspective, skin represents a highly exposed target for chemicals and toxins. For food contaminants in general and for mycotoxins in particular, dermal exposure through contaminated food commodities during harvest and processing is becoming a topic of great interest (Doi and Uetsuka 2014), and it poses concerns in addition to the classical oral administration route (Katrine et al. 2017). Among food contaminants, deoxynivalenol (DON, vomitoxin) is one of the most commonly detected mycotoxins produced by the *Fusarium species complex* (Gruber-Dorninger et al. 2016; van der Lee et al. 2015). DON is classified in the trichothecene structural group and acts at molecular level via inhibition of protein synthesis (Cundliffe et al. 1974; Ueno 1977). This event is initiated by binding to the A-site of the 60S large ribosomal subunit (Dellafiora et al. 2017; Garreau de Loubresse et al. 2014) and ultimately leads to the impairment of the peptidyl-transferase activity of the organelle. In line, the toxin extensively impairs cellular functions, and this is associated with organ-specific phenotypic manifestations (Pestka 2010a, b). As example, DON is known to impair the intestinal barrier system; it targets cell–cell-junctional proteins and mucous layer hampering in this way gut functionality (Beisl et al. 2020, 2021; Pinton and Oswald 2014; Robert et al. 2017; Wang et al. 2020).

At cutaneous level, DON sustains pro-inflammatory reactions and activates the AP-1 and NF-κB signaling cascade in vitro (Mishra et al. 2014). On these molecular premises, in vivo, DON can act as skin tumor initiator (Mishra et al. 2016) and this response is sensitive to the combined treatment with the antioxidant *N*-acetyl-cysteine and the anti-inflammatory drug celecoxib (Mishra et al. 2020a). However, much remains to be elucidated to understand the mechanisms sustaining the dermotoxic effects of DON. For instance, it was recently described how even oral intake could enhance inflammatory response in allergic dermatitis (Aihara et al. 2020). In line, accurate definition of molecular events triggered by DON is of crucial importance for the assessment of the risk associated with the cutaneous exposure through contaminated food commodities.

Epidermoid carcinoma cells A431 are routinely used as skin-derived cell model (Li et al. 2016; Smina et al. 2015). We recently demonstrated that DON impairs the capability of A431 to respond to uniaxial cell stretching and that the toxin targets several proteins essential for the maintenance of cell adhesion and morphology (Del Favero et al. 2018a). This is particularly relevant since biomechanical plasticity and adaptive response to movement are obvious essential components of the skin barrier functionality. In this study, we applied a combinatory microscopy and proteomics approach to unravel the molecular events, downstreaming from the ribosomal inhibition, toward the loss of membrane structural integrity. Proteomics profiling combined with phosphoproteomics was used to characterize the molecular signature of DON on A431 cells and to highlight the steps leading to the alteration of lipid biosynthesis machinery. Ultimately, to sustain the toxicological relevance of our findings also in non-transformed cells, we compared the effects of DON with human primary keratinocytes (HEKn) regarding proteome alterations and post-translational modifications.

## Materials and methods

### Cell culture

Epidermoid carcinoma cells A431 were cultivated as previously described (Del Favero et al. 2018a) in Minimum Essential Medium (MEM) with l-glutamine (4.5 g/L), 10% (*v/v*) heat-inactivated fetal bovine serum (FBS) and 1% (*v/v*) penicillin/streptomycin and maintained in controlled humidified incubators at 37 °C and 5% CO_2_. Primary Epidermal Keratinocytes (HEKn; Normal, Human, Neonatal Foreskin ATCC® PCS-200-010™) were cultivated according to the specification of the supplier in Dermal Cell Basal Medium (ATCC® PCS-200-030™) including Keratinocyte Growth Kit (ATCC® PCS-200-040™). If not otherwise specified, cell culture reagents were purchased from GIBCO Invitrogen (Karlsruhe, Germany), Sigma-Aldrich Chemie GmbH (Munich, Germany), Sarstedt AG & Co (Nuembrecht, Germany), VWR International GmbH (Vienna, Austria) and Thermo Fisher Scientific GmbH (Vienna, Austria). Commercially available DON was purchased from Romer Labs (Tulln, Austria). Solid substance was dissolved in dimethyl sulfoxide (DMSO; Carl Roth GmbH, Karlsruhe, Germany) and diluted in cell culture media (1:1000). Respective DMSO concentration (0.1%) was used as negative/solvent control (controls, CONT).

### Cell fractionation

To obtain the cytoplasmic fraction, cells were lysed in isotonic lysis buffer (10 mM HEPES/NaOH, pH 7.4, 0.25 M sucrose, 10 mM NaCl, 3 mM MgCl2, 0.5% Triton X-100) supplemented with Protease and Phosphatase Inhibitor Cocktail (Sigma-Aldrich, Vienna, Austria) and 1 mM PMSF under mechanical shear stress. By centrifugation at 3500 g and 4 °C for 5 min, the cytoplasmic proteins were separated from the nucleic fraction and precipitated overnight with ice-cold ethanol at − 20 °C. The remaining nuclei were thoroughly resuspended in TE–NaCl and TE–NP-40 buffer and incubated on ice. After centrifugation, the supernatant was precipitated in ethanol overnight.

### Sample preparation

(i) In-gel digestion of A431 cell fractions: 50 μg of each sample were loaded on an SDS-PAGE and allowed to enter the separation gel for 0.3 cm. After that, proteins in the gel were stained by an MS-compatible silver staining procedure and the total protein amount was subjected to an established in-gel digestion protocol (Bileck et al. 2014). Upon reduction with DTT and alkylation with IAA, the proteins were digested in a two-step protocol for 14 h overnight and 4 h at 37 °C using Trypsin/Lys-C Mix (MS grade; Promega Corporation, Madison, WI). The gel pieces containing the digested peptides were extracted with an extraction buffer (1:1 mixture of 5% formic acid/ACN).

(ii) Filter-aided sample preparation of HEKn cell fractions: protein fractions were subjected to a filter-assisted proteolytic digestion with a modified version of the FASP protocol (Bileck et al. 2014; Wisniewski et al. 2009). In short, 20 µg of proteins were loaded onto a pre-wetted MWCO filter (Merck KGaA, Darmstadt, Germany) with a pore size of 10 kD, followed by reduction of disulfide bonds with dithiothreitol (DTT), alkylation with iodoacetamide (IAA) and washing steps with 50 mM ammonium bicarbonate buffer. Digestion of proteins was achieved by applying two times Trypsin/Lys-C with Mass Spec Grade quality (Promega, Mannheim, Germany), at first overnight, and in a second step for 4 h. Resulting peptides were eluted through the filter by centrifugation.

(iii) Digestion protocol with the S-trap technology (Protifi, LLC., New York; USA): 50 µg or 75 µg protein solubilized in buffer containing 5% SDS were reduced and alkylated as described above. After addition of trapping buffer (90% vol/vol Methanol, 0.1 M Triethylammonium bicarbonate) samples were loaded onto the cartridges and digested with Trypsin/Lys-C Mix at 47 °C for one hour. Supernatants containing the collected peptides were dried before instrumental analysis.

### Phosphopeptide enrichment via metal oxide affinity chromatography (MOAC)

Enrichment was performed with tryptic digests of cytoplasmic fractions of A431 and HEKn cell lines totaling 50 µg and 75 µg, respectively. For optimal peptide recovery and purity of the samples, the digestion protocol with the S-trap technology was employed. The peptides were resuspended in Binding Buffer containing 1 M glycolic acid, 5% (vol/vol) trifluoroacetic acid (TFA), 80% (vol/vol) LC–MS grade acetonitrile and loaded onto TiO_2_ Mag Sepharose beads (GE Healthcare GmbH, Solingen, Germany) preconditioned with Binding Buffer. After 30 min incubation at room temperature and three subsequent washing steps (80% vol/vol acetonitrile, 1% vol/vol TFA), phosphopeptides were eluted with 5% (vol/vol) ammonium hydroxide solution. Supernatants containing the collected peptides were dried before instrumental analysis.

### LC–MS/MS analysis

Dried samples were reconstituted in 5 µL 30% FA containing 10 fmol each of four synthetic standard peptides and diluted with 40 µL mobile phase A (98% H_2_O, 2% ACN, 0.1% FA). 5 µL of this solution was then injected into a Dionex Ultimate 3000 nano LC-system coupled to a QExactive orbitrap mass spectrometer equipped with a nanospray ion source (Thermo Fisher Scientific, Austria). As a pre-concentration step, peptides were loaded on a 2 cm x 100 µm C18 Pepmap100 pre-column (Thermo Fisher Scientific, Austria) at a flow rate of 10 µL/min using mobile phase A. Elution from the pre-column to a 50 cm ×  75 µm Pepmap100 analytical column (Thermo Fisher Scientific, Austria) and subsequent separation was achieved at a flow rate of 300 nL/min using a gradient of 8–40% mobile phase B (80% ACN, 2% H_2_O, 0.1% FA) over 90 min. For mass spectrometric detection, MS scans were performed in the range from *m*/*z* 400–1400 at a resolution of 70,000 (at *m*/*z* = 200). MS/MS scans of the 8 most abundant ions were achieved through HCD fragmentation at 30% normalized collision energy and analyzed in the orbitrap at a resolution of 17,500 (at *m*/*z* = 200).

### MS data processing

Identification of proteins as well as label-free quantification (LFQ) and statistical analyses were performed using the MaxQuant 1.6.0.1 software (Cox and Mann 2008) including the Andromeda search engine (Cox et al. 2011) and the Perseus statistical analysis package, a commonly used workflow for processing and statistical assessment of shotgun proteomics data (Mayer et al. 2018; Tyanova et al. 2016). Proteins were identified using the UniProt database for human proteins (version 03/2018, restricted to reviewed entries only with 20,316 entries), a peptide mass tolerance of 25 ppm, an MS/MS match tolerance of 20 ppm and a maximum of two missed cleavages with trypsin as protease. Search criteria further included carbamidomethylation of cysteines as fixed modification, methionine oxidation as well as N-terminal protein acetylation as variable modifications, and a minimum of two peptide identifications per protein, at least one of them unique. Furthermore, match between runs was performed using a 1-min match time window and a 15-min alignment time window. For both, peptides and proteins, a false discovery rate (FDR) of less than 0.01 was applied; the FDR was determined by the target-decoy approach using the reversed version of the database as decoy. To determine protein groups that were significantly up- or down-regulated upon treatment, Perseus statistical analysis package was used and differences of LFQ values were calculated. LFQ values of technical duplicates were averaged and the biological replicates considered as independent. Common contaminants were removed. Changes in protein abundance values between treated and untreated cells were determined by a two-sided t test with an FDR < 0.05 and setting S0 (x-value of the hyperbolic tangent of the function separating significant events) to 0.1. Principle component analysis (PCA) results are provided in supplementary Figures S1–S2 and Figures S3–S4 for A431 and HEKn cells respectively. Data derived from bioinformatic analysis (oPOSSUM software (Ho Sui et al. 2007; Ho Sui et al. 2005; Kwon et al. 2012)) of up- and down-regulated proteins can be found in Supplementary Table 1. For the analysis of individual phosphopeptides, the software Peaks (Peaks Studio 10.0 build 20190129) was used. For the database supported search, a version of the human proteome from uniprot with 20.429 entries (October 2019) was employed. Dynamic modifications, namely N-terminal acetylation, methionine oxidation, arginine deamidation, and phosphorylation of serine, threonine and tyrosine, were taken into account. Carbamidomethylation of cysteine was considered a static modification. The maximal mass deviation of the precursor peptide was 15 ppm; the maximal fragment mass deviation was set to 0.05 Da. For phospopeptide identification, a strict FDR of 1% was applied. For comparative analysis, a significance threshold of 15 according to Peaks and a minimum of twofold change on average were defined.

Kinase-substrate enrichment analysis was performed on site-centric quantification data obtained via MaxQuant data processing as previously described (Weiss et al. 2021). Search parameters were adjusted to additional dynamic modification of phosphorylation of serine, threonine and tyrosine. Enrichment analysis was performed on Class 1 phosphosites (*p* > 0.75) utilizing PhosphoSitePlus and NetworKIN, applying a NetworKIN score cutoff of 2, *p* value cutoff of 0.05 and substrate count cutoff 3.(Casado et al. 2013; Horn et al. 2014; Hornbeck et al. 2014).

### Immunofluorescence

Immunolocalization experiments were performed as previously described with minor modifications (Del Favero et al. 2020). A431 cells were incubated for 24 h or 6 h hours with DON (0.1–1–10 µM) or with solvent control. At the end of the incubation, cells were fixed with pre-warmed formaldeyde (3.7%, 37 °C) and permeabilized with 0.2% Triton-X100. Blocking was perfomed with 1% Donkey serum (1 h) and targets of interest were recognized with anti-KLF4 mouse monoclonal antibody (56CT5.1.6_ab75486, Abcam), anti TOM20 mouse monoclonal antibody (F-10_sc-17764, Santa Cruz). After multiple washig steps, species-specific secondary antibodies were applied. This includes Alexa Fluor 568 Donkey anti-Rabbit (A10042) and Alexa Fluor 647 (A31571) Donkey anti-Mouse. Actin was counterstained with Alexa Fluor™ 488 Phalloidin (all from Molecular Probes, Life Technologies, Thermo Fisher Scientific, Waltham, USA). The slides were rinsed and post-fixed with 3.7% formaldehyde (10 min, RT); at the end of the post-fixation, 100 mM glycine was used to mask reactive sites and slides were mounted and sealed with Roti-Mount FluoCare (Roth, Graz, Austria) with DAPI. Confocal images were acquired with a Confocal LSM Zeiss 710 equipped with ELYRA PS. 1 system and alpha plan apochromat 100X/1.46 Oil DIOC M27 objective. Image analysis was performed with ImageJ software on *n* > 50 ROI (regions of interest) randomly selected from images acquired from 3 independent datasets. Klf4 and TOM20 signals were quantified as data expressed as relative fluorescence units in comparison to controls.

### Live cell imaging

For live cell imaging acquisition, CellMask™ Deep Red Plasma membrane stain (1:1000 dilution, depicted in white) was used. Cell nuclei were counterstained with Hoechst 33258 (1:1000 dilution, depicted in blue). Staining solutions were diluted in Live Cell Imaging Solution (all from Molecular Probes, Life Technologies, Thermo Fisher Scientific, Waltham, USA). At the end of the incubation time, cells were washed and pre-warmed Live Cell Imaging Solution was used for the microscopy experiment. Time series were acquired with a Confocal LSM Zeiss 710 equipped with ELYRA PS. 1 using a Plan Apochromat 63X/1.4 oil objective. Filopodia were quantified using the plugin FiloQuant for ImageJ. The cell edges and filopodia were determined for each picture either by automated analysis cross checked by step-by-step analysis to achieve representative outcomes. The quantification (Fig. 5d–h) was carried out as described by Jacquemet et al*.* (2017) with small adaptation. Filopodia density was calculated from the skeletonized images as the ratio of filopodia number and cell edge length for each individual picture. Analyzed datasets derived from 3 independent cell preparations.

### Membrane fluidity assay

Membrane fluidity was measured adapting to the protocols from Zhang et al*.* (2011) and Del Favero et al*. *(2018b). Briefly, cells were either pre-incubated with the toxin (DON 24 h 0.1–10 μM) or challenged with the mycotoxin after the incubation with 1-pyrenedecanoic acid (PDA; Sigma Aldrich, 37 °C 1 h). Measurements were performed with Cytation3 Imaging Multi-Mode Reader (BioTek, Winooski, VT, USA) using 344 nm excitation wavelength and measuring emission at 375 nm (PDA monomeric form) and at 470 nm (PDA excimeric form). Cholesterol complexing agent methyl-beta cyclodextrin (MβCD, 10–100 µM) and H_2_O_2_ (100–1000 µM) were included as positive controls.

### Experimental design and statistical rationale

**Fig. 1:**
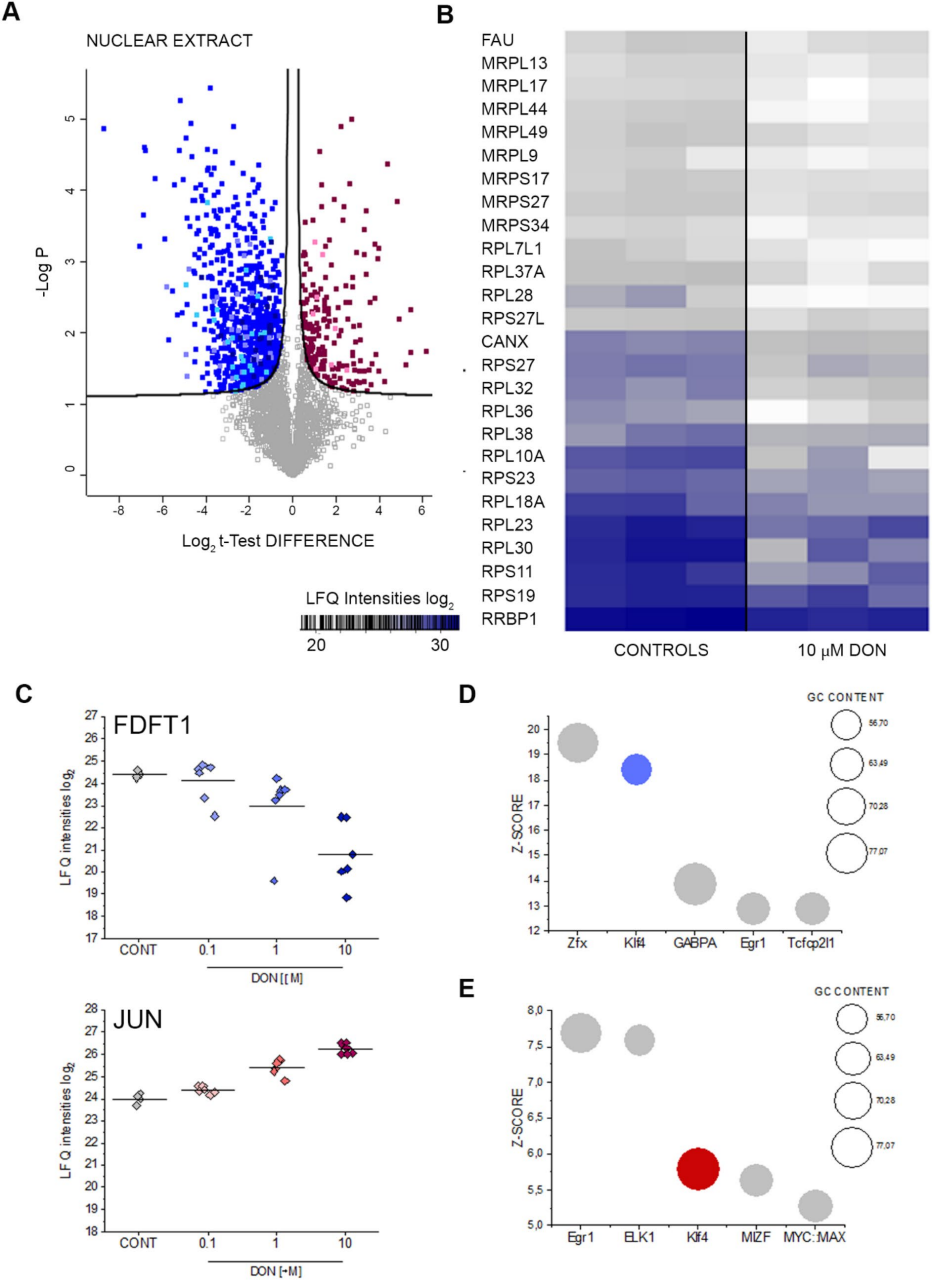
**a** Volcano plots depicting significant protein regulation (blue down-regulated and red up-regulated) between controls (CONT) and 10 µM DON (Nuclear Extract). Nuclear pores proteins (rose), Mitochondrial proteins (light blue), Ribonuclear proteins (violet), Ubiquitin/Proteasome (dark blue). **b** Ribosomal proteins affected by 24 h incubation with DON. **c** Concentration dependent effect of DON on squalene synthase (FDFT1) and transcription factor AP-1 component (JUN). Transcription factors associated with the proteins significantly down (**d**) and up (**e**) regulated after incubation with 10 µM DON identified by oPOSSUM search (Kwon et al. 2012)

To support statistical analysis, proteomic analyses were performed on the practical minimum of three biological replicates measured in technical duplicates per cell state (solvent controls, 0.1 µM, 1 µM and 10 µM DON, Fig. 1c, Supplementary Fig. S5). Cytoplasmic fractions and nuclear extracts were analyzed separately. For the control group of the nuclear extract fraction (Fig. 1c, Supplementary Fig. S5) one technical replicate failed and was omitted. For the identification of proteins, a false discovery rate of 0.01 was applied both at peptide and protein level. For the calculation of significant alterations of protein abundance values, a permutation-based false discovery rate calculation applying FDR < 0.05 was applied to *t* tests of LFQ mean values. Consensus binding sites determined using the oPOSSUM software were considered significant when exceeding mean values with the addition of two standard deviations. For the selection of significantly regulated phosphopeptides, a significance threshold of a testing *p* value < 0.05 was applied.

**Fig. 2:**
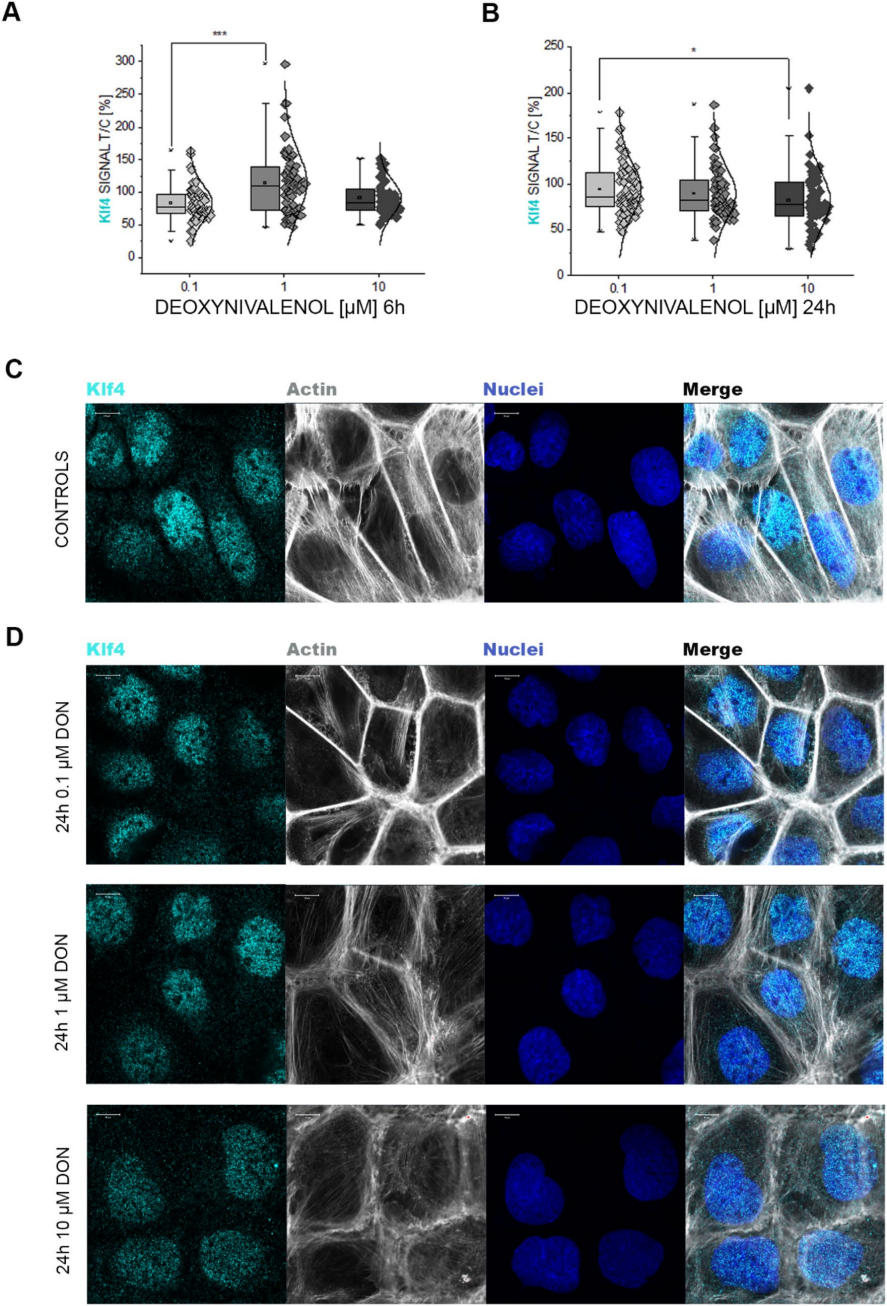
Immunofluorescence localization of Klf4 in A431 cells. **a** quantification of the Klf4 signal at nuclear level after 6-h incubation and **b** 24 h incubation. Data results from the quantification on n ≥ 50 ROI randomly selected from 3 independent experiments. * p < 0.05 and *** p < 0.001 at Student’s *t* Test. **c** Representative images of the immunolocalization of Klf4 (light blue) and **d** after 24 h incubation with DON (0.1, 1 and 10 μM). Actin cytoskeleton is counterstained with phalloidin (depicted in gray) and cell nuclei with dapi (depicted in blue)

For the data presented in Figs. 2 and 4, image analysis is the result of the quantification of at least 6 different optical fields obtained from at least three independent experiments. For the membrane fluidity assay, data are expressed as mean of 7 individually conducted experiments, comprising three technical replicates. Statistical analysis was performed applying one-way ANOVA test followed by Fisher test for pairwise comparisons (threshold values *p* < 0.05, Origin Pro 9.1G; OriginLab, Northampton, USA) or with Student’s *t* Test. Graphical representation of GO terms representing biological processes and cellular components was obtained with Origin 2018b with data obtained using the DAVID Bioinformatics Resources (Huang da et al. 2009) submitting all proteins significantly up- or down-regulated, respectively. Cell lines are compared directly for the same Cellular Component or Biological Process GO Terms indicating in red the data derived from up-regulated proteins and in blue the down-regulated ones. Data used for this representation are provided in Supplementary Material Table 4.

## Results

### DON induced proteome alterations in A431 cells point toward regulation of transcription factor KLF4

Incubation of A431 cells with 10 µM DON triggered significant alterations of the proteome profile of the epidermoid cells. While data regarding cytoplasmic proteins were published previously (Del Favero et al. 2018a), here, we focused on nuclear extracts while adding novel data regarding the cytoplasmic proteins based on independent measurements. Out of a total of 3363 identified proteins in the nuclear extracts, 988 proteins were found significantly regulated (Fig. 1a, FDR 0.05). Among these, several ribonuclear proteins were down-regulated (Fig. 1a, b), thus confirming the ribosome as a main molecular target for DON (Cundliffe et al. 1974; Ueno 1977). Remarkably, downregulation of the ubiquitin/proteasome pathway as well as mitochondrial proteins crucial for oxidative phosphorylation (OXPHOS) was also observed (Fig. 1a) in addition to several proteins mediating lipid synthesis. To assess the specificity of this effect, dose–response experiments were performed measuring the effect of increasing concentrations of the toxin in the nuclear fraction (Fig. 1c, Supplementary Fig. S5) as well as in the cytoplasmic compartment (Supplementary Fig. S5, unreported data extracted from a previously published dataset (Del Favero et al. 2018a)). Squalene synthase (FDFT1) was found significantly regulated in a dose-dependent fashion in both subcellular fractions (Fig. 1c nuclear extracts, Supplementary Fig. S5A cytoplasmic fraction). Similar regulatory pattern could be observed for the lysosomal acid lipase/cholesteryl ester hydrolase (LIPA) and the farnesyl pyrophosphate synthase (FDPS), the mitochondrial methylmalonyl-CoA mutase (MUT, cytosolic fraction) and interferon-induced transmembrane protein 3 (IFITM3; cytosolic fraction), which were all regulated in a dose-dependent manner (Supplementary Figure S5C-H). Remarkable was also the concentration-dependent effect of the toxin on the transcription factor AP-1 (JUN, Fig. 1c) and on WWTR1 (WW domain-containing transcription regulator protein 1, Supplementary Fig. S5B). WWTR1, as component of the Hippo pathway, has been already associated with the regulation of the complex machinery governing cell adhesion and perception of mechanical forces, as well as to the development of some skin cancer types (Andl et al. 2017). Bioinformatics processing of the data with oPOSSUM software (Kwon et al. 2012) (Nuclear extracts, Fig. 1d and e, Supplementary Material Table 1) revealed the transcription factor krueppel-like factor 4 (Klf4) as a common denominator of the regulatory events triggered by DON in A431 cells. Klf4 was previously described for its role in the maintance of skin barrier function (Segre et al. 1999), hence its involvement is of great relevance for the comprehension of the dermotoxic potential of DON. Taking this as a starting point, we decided to confirm the effect of the mycotoxin on Klf4 with an independent workflow. Indeed, also with immunofluorescence and confocal imaging we could observe a concentration- and time-dependent effect of DON on the subcellular distribution of Klf4. DON increased Klf4 localization in the nuclear region after 6-h incubation (Fig. 2a) which was followed by a decrease at a longer incubation time (24 h, Fig. 2b–d).

### Unfolded protein response mitochondrial induced by DON

**Fig. 3:**
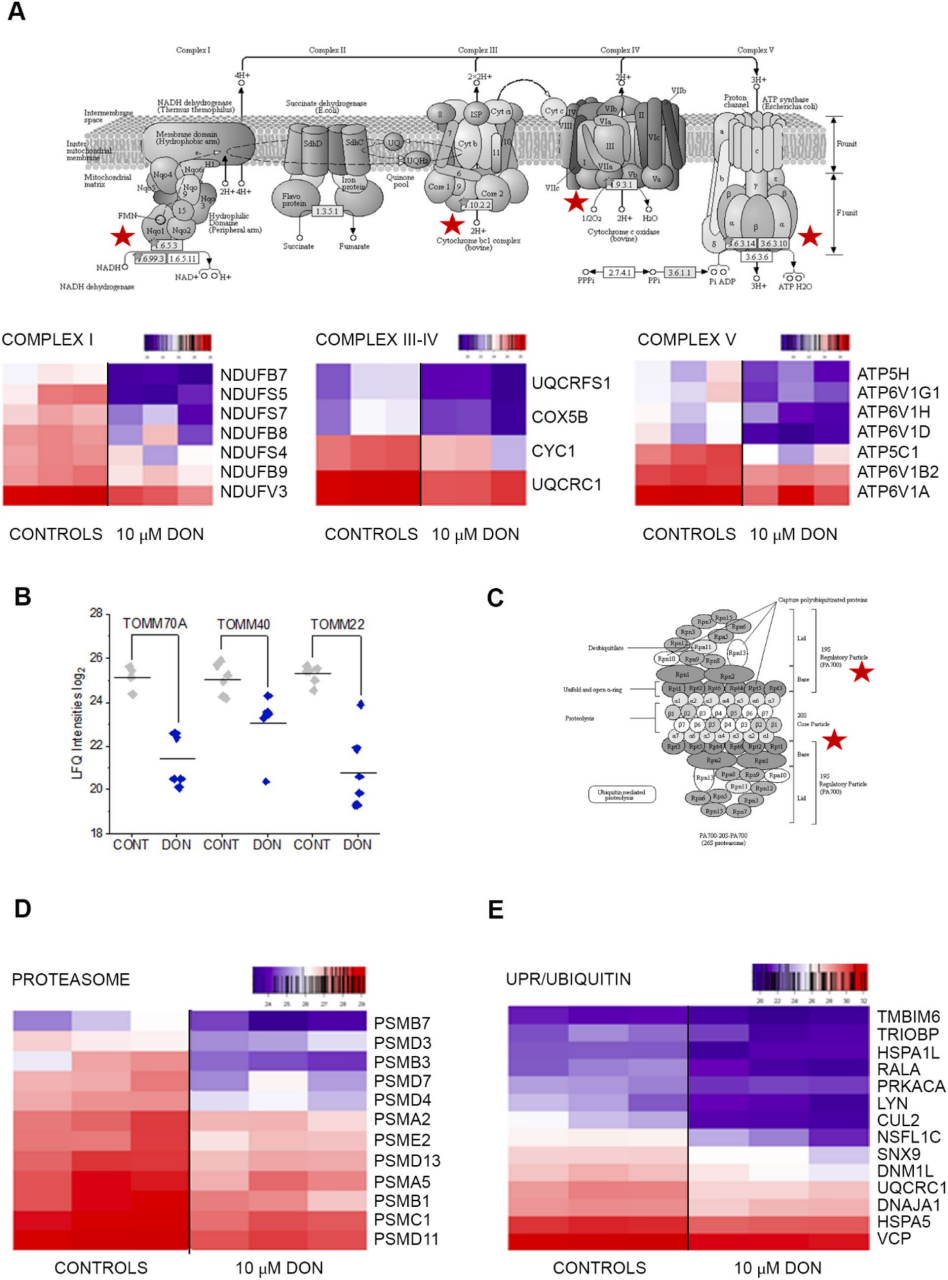
Effect of DON at mitochondrial level. **a** Mitochondrial respiratory chain subunits altered by incubation with DON. Images are generated from KEGG database (Kanehisa et al. 2016, 2017; Kanehisa and Goto 2000) and reused modified with permission [KEGG Copyright Permission 200848]. Heat maps depict the proteins of the COMPLEX I, III–IV and V targeted by DON. **b** Effect of DON (blue) on the mitochondrial import proteins TOMM22/40 and 70A in comparison to controls (CONT, gray). **c** Proteasome subunits altered by incubation with DON. Images are generated from KEGG database (Kanehisa et al. 2016, 2017; Kanehisa and Goto 2000) and reused modified with permission [KEGG Copyright Permission 200848]. Red Stars indicate the subunits targeted by the mycotoxin. Heat maps depict the proteins of the proteasome (**d**) and of the ubiquitin complex (**e**) regulated by incubation with DON

**Fig. 4:**
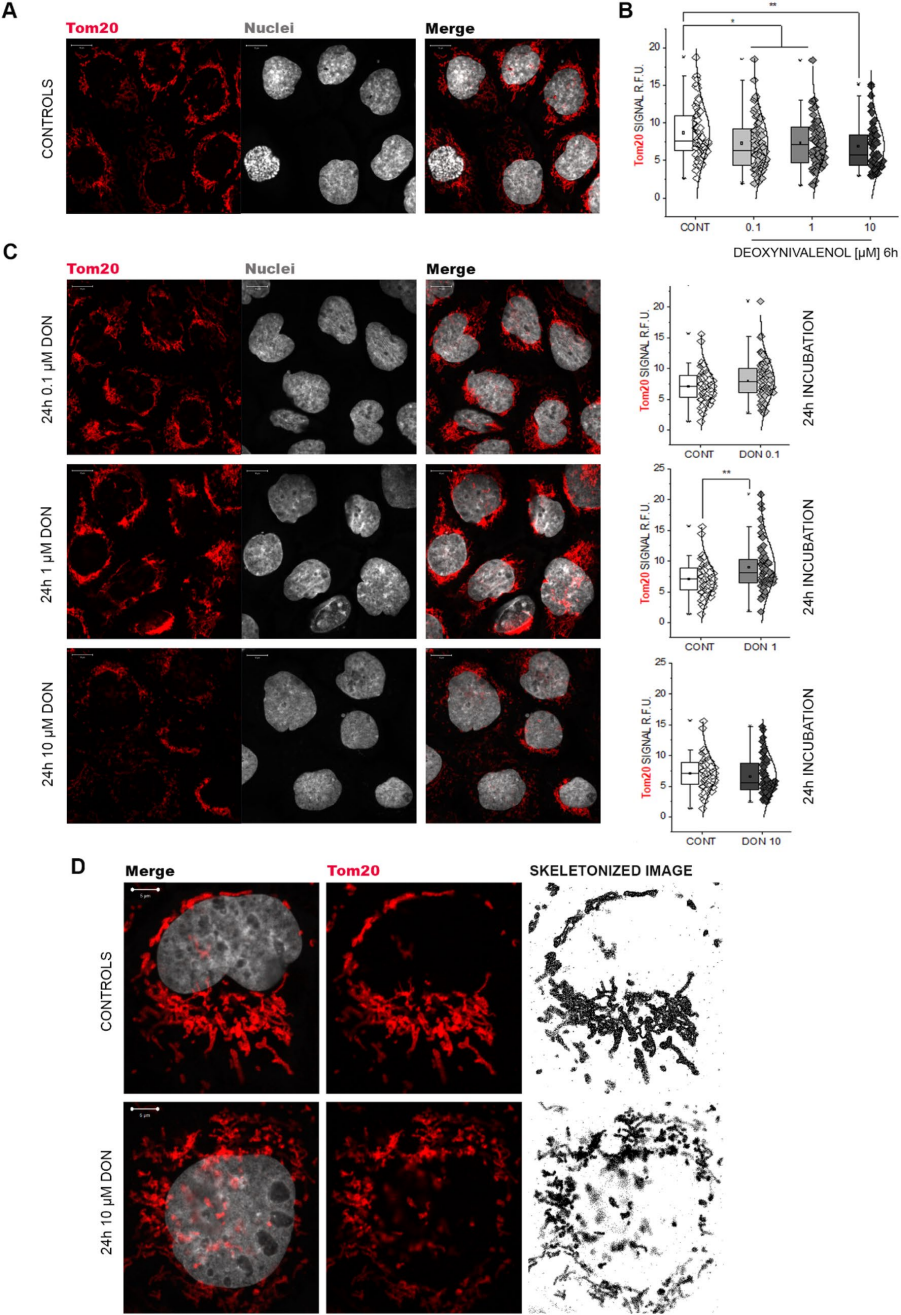
Immunofluorescence localization of Tom20 in A431 cells. **a** appearance of the mitochondrial protein in control conditions. **b** Tom20 signal quantification after incubation with DON for 6 h. **c** Morphology and signal quantification of Tom20 after incubation with DON for 24 h. Data results from the quantification on n ≥ 50 ROI randomly selected from 3 independent experiments, *p < 0.05 and **p < 0.01 at Student’s *t* Test. **d** Detail of the morphological changes triggered by 10 μM DON (24 h incubation) on the mitochondrial network as compared to controls. Cell nuclei are counterstained with dapi (depicted in gray)

In addition to its role in sustaining skin barrier function, Klf4 plays a central role in the regulation of metabolism and in particular mitochondrial homeostasis (Tung and Xia 2018). In line, among the most specifically challenged organelles after DON treatment, mitochondria were outstanding. More than 20 mitochondrial proteins were found significantly down-regulated (Fig. 3). Among these, there were seven members of the accessory subunits of the NADH dehydrogenase (Complex I), and four proteins of the cytochrome complex (Fig. 3a). Moreover, 7 proteins constituting the ATP synthase complex were also significantly regulated (Fig. 3a), thus confirming the central role of mitochondria and mitochondrial stress in the mechanism of action of DON (Bin-Umer et al. 2014; Ren et al. 2020). In addition to respiratory chain proteins, three mitochondrial import proteins (Wiedemann et al. 2003), namely TOMM70A, TOMM40, TOMM22 (Fig. 3b) were found significantly down-regulated. Incubation with DON reduced also the abundance of 12 protein forming the proteasome complex (Fig. 3c, d) and further 14 proteins related to ubiquitination processes (Gene Ontology), indicating overall an impairment of the protein turnover apparatus (Fig. 3e). Among these, there was bax inhibitor 1 (TMBIM6; Fig. 3e), a cytoprotective protein that exerts its function through the modulation of UPR pathway (Krajewska et al. 2011), the DnaJ homolog subfamily A member 1 (DNAJA1; Fig. 3e) that regulates protein import in the mitochondria (Radons 2016) and the heat shock protein 70 kDa like 1 (HSPA1L) that upon ATP hydrolysis allows the elimination of damaged proteins (Radons 2016). In light of the capability of DON to trigger a proteome adaption retracing the UPR-mitochondrial, we also assessed if this was mirrored in the structure of the organelles. It is well known that mitochondrial functional/energetic status is tightly related to morphology and distribution underpinning the fusion–fission equilibrium (Wakim et al. 2017). In agreement with the proteome profiling, we observed that DON dose-dependently reduced the signal generated by the immunolocalization of TOM20 (Fig. 4a and b). Moreover, after 24 h incubation, DON induced a concentration dependent disorganization of the mitochondrial network. This was visible as progressive accumulation of TOM20 signal in the perinuclear region (Fig. 4c) and culminating in almost complete fragmentation upon incubation with 10 µM DON (Fig. 4d).

### Impact of DON on membrane function

**Fig. 5:**
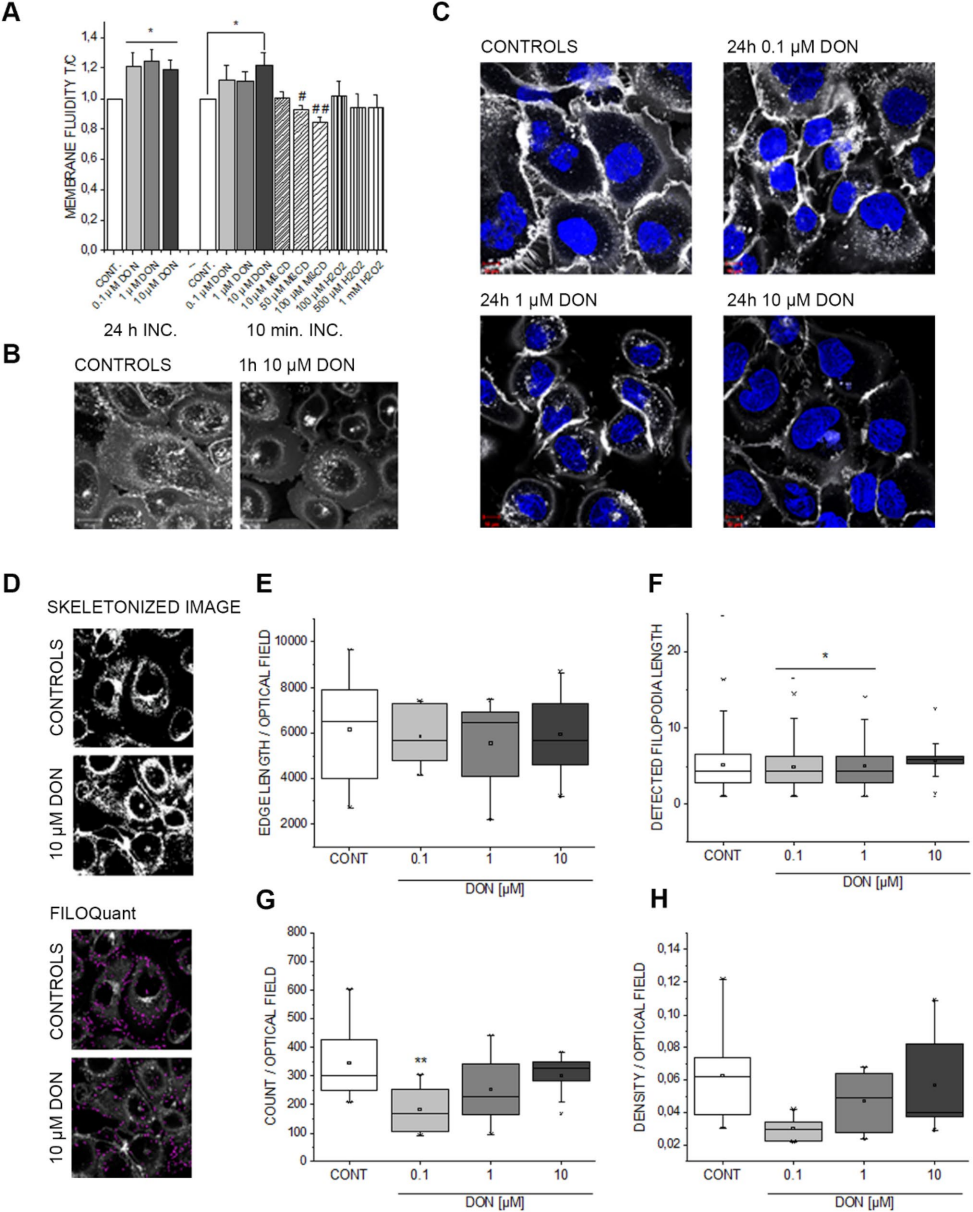
Effect of DON on A431 membrane. **a** Changes in membrane fluidity after 24 h incubation and 10-min exposure to DON, methyl-beta cyclodextrin (MβCD) and H_2_O_2_. **b** Representative pictures of cell membrane appearance after 1-h incubation with or without DON (10 µM, scale bars 20 µm). **c** Concentration dependent effect of DON on cell membrane morphology after 24 h incubation (CellMask in white, Hoechst 33258 in blue) scale bars 10 µm. **d** Appearance of the images during the step-by-step analysis of filopodia using FiloQuant. **e** Edge lengths per optical field expressed as pixels of each picture. **f** Average filopodia lengths (pixels; n > 1000 filopodia/condition, * p < 0.05, Student’s *t* Test). **g** Filopodia count per optical field (**p < 0.01, One way ANOVA). **h** Filopodia density expressed as filopodia number per edge lengths per optical field. Data are mean of at least 6 independent optical fields

Overall, both untargeted (proteomics) and targeted (microscopy) analysis supported the interpretation that DON-associated dermotoxicity could involve Klf4-related pathways. Once having ascertained the effect on mitochondria, we moved to verify other essential downstreaming pathways related to the activation of the transcription factor and we focused our attention on barrier function. Particularly, working with in vitro models, this can reflect on cell membrane structure and biophysical properties. DON exerted a time- and concentration-dependent effect on membrane fluidity of A431 cells (Fig. 5a). H_2_O_2_ added as pro-oxidant challenge failed to reproduce the effect triggered by the toxin. The cholesterol complexing agent MβCD (positive control; Fig. 5a) induced a concentration dependent decrease of the membrane fluidity of A431, confirming the performance of the assay with our cell model. Morphological evaluation of the cell membrane obtained through live cell imaging revealed prominent changes in cell membrane appearance after 1-h incubation with the toxin (10 µM; Fig. 5b) and a clear concentration-dependent effect after 24 h of incubation (Fig. 5c). Decrease of the staining intensity was accompanied by the appearance of areas of uneven accumulation of the membrane dye (CellMask), suggesting massive changes in membrane dynamics. To provide a preliminary assessment of these modulations, images were analyzed following the FILOquant workflow (Fig. 5d). Software analysis revealed homogeneous edge length in the images (Fig. 5e) and a significant decrease of the average filopodia length for the cells incubated with the lowest concentrations of the toxin (Fig. 5f; 0.1 µM DON vs CONT *p* = 0.014; 1 µM DON vs CONT *p* = 0.043 Student's *t* Test). Of note, an increase was detected for cells incubated with the highest concentration of DON. However, the performance of the evaluation software in this case is questioned by the apparent decrease of the CellMask signal, as well as by the uneven accumulation of the dye in cell-junctional areas. Moreover, data showed an alteration in the distribution of filopodia count (Fig. 5g, 0.1 µM DON) and a tendency on the decrease of the filopodia density (Fig. 5h).

To rule out if the effects of the mycotoxin on the cell membrane and cell membrane dynamics were associated with unspecific oxidative stress, intracellular ROS levels were determined by DCF-DA assay (Supplementary Figure S6A). Indeed, no increase in intracellular ROS levels was observed in A431 cells after cellular exposure to the toxin (max. 180 min.; Supplementary Fig. S6A). After 24 h, the most relevant proteins typical for an oxidative stress signature, namely peroxiredoxins (Supplementary Fig. S6B nuclear extract NE and cytosolic fraction CYT), and representative members of glutathione pathway (Supplementary Fig. S6C cytosolic fraction CYT), were not regulated. However, a significant decrease/depletion in the thioredoxins pools in both nuclear and cytosolic compartment was observed (Supplementary Fig. S6D nuclear extract NE and cytosolic fraction CYT).

### Effect of DON on primary epidermal keratinocytes

**Fig. 6:**
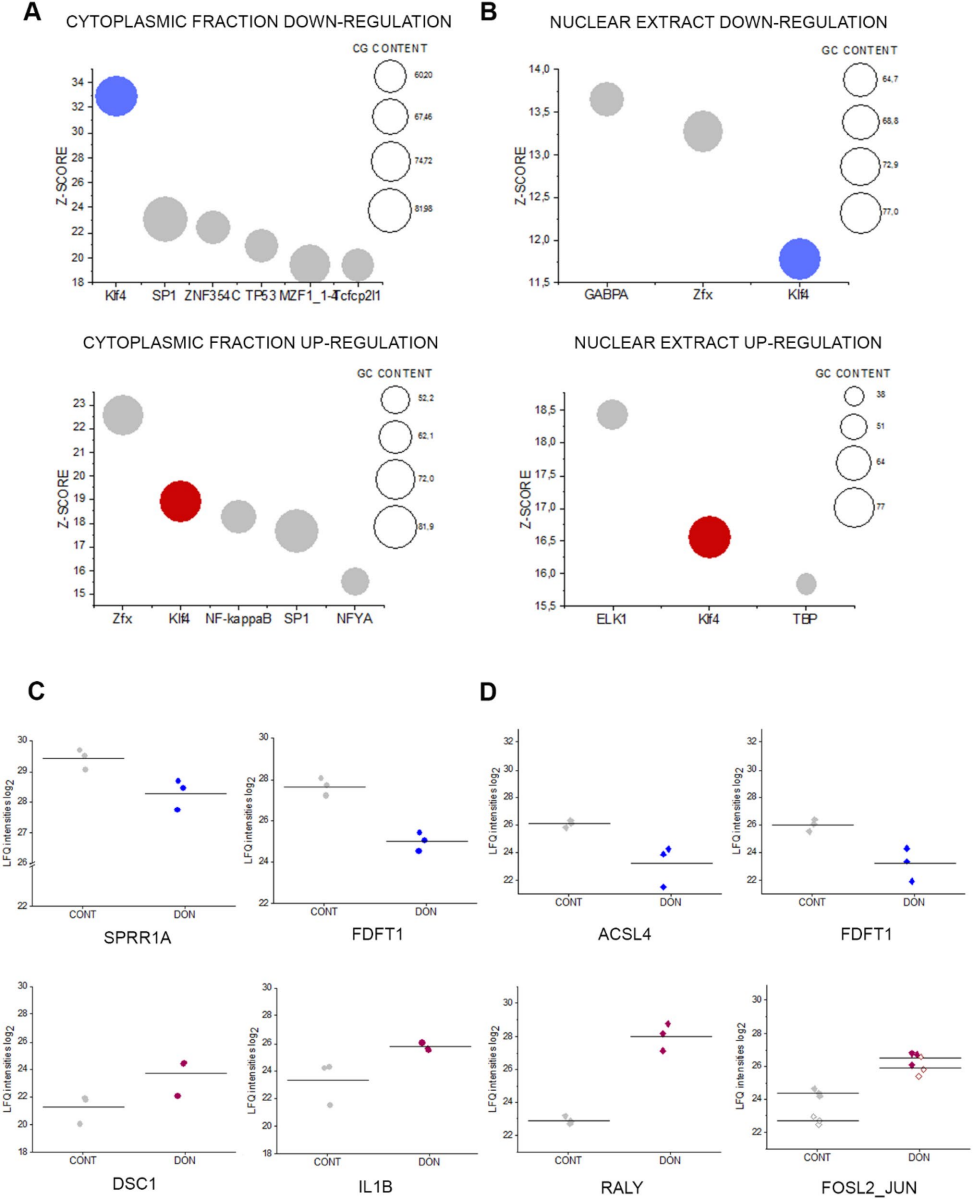
Significant protein regulation between controls (CONT) and 10 µM DON in HEKn cells. Transcription factors associated with the proteins significantly up- and down-regulated after incubation with 10 µM DON identified by oPOSSUM Search (Kwon et al. 2012) in the cytoplasmic fraction (**a**) and nuclear extracts (**b**). **c** Selected regulated proteins in the cytoplasmic fraction: cornifin-A (SPRR1A), squalene synthase (FDFT1), desmocollin-1 (DSC-1), interleukin-1 beta (IL1B). **d** Selected regulated protein in the nuclear extract: long-chain-fatty-acid–CoA ligase 4 (ACSL4) and squalene synthase (FDFT1) RNA-binding protein Raly (RALY), Fos-related antigen 2 (FOSL2, full diamonds) and transcription factor jun-D/JUND (JUN, empty diamonds)

To expand the toxicological relevance of our findings, namely the involvement of proteins regulating cell membrane functions/barrier integrity as crucial targets downstreaming from DON-induced ribosomal inhibition, we repeated the experiments on human primary epidermal keratinocytes (HEKn). 24 h incubation with 10 µM DON induced significant regulation on more than 600 proteins out of 2977 identified proteins in the cytoplasmic fraction and more than 1600 out of 3023 identified proteins in the nuclear fraction (FDR 0.05, Supplementary Fig. S7). Bioinformatics data analysis indicated once again the transcription factor Klf4 as a key player in DON-induced response (Fig. 6a and b). In line, several proteins underpinning an involvement of skin barrier function were found disregulated (Fig. 6c, d, Supplementary Fig. S7). Similar to the A431, the squalene synthase (FDFT1) was significantly down-regulated also in the HEKn model (Fig. 6c). Among the other down-regulated proteins, cornifin-A (SPRR1A, Fig. 6c) appeared particularly meaningful in light of its function as cross-linking envelope protein for the keratinocytes (Rajagopalan et al. 2018). Similarly related to epithelial integrity were also syndecan-1 (SDC1) and galectin-3-binding protein (LGALS3BP) which mediate cytoskeleton–matrix and cell–cell adhesion (Carulli et al. 2012; Fortuna-Costa et al. 2014) (Supplementary Fig. S7C). Among the up-regulated proteins in the cytoplasmic fraction, significant regulation was observed for structural proteins like desmocollin-1 (DSC1) and keratin 18 (KRT18) (Fig. 6c and Supplementary Fig. S7C). Remarkably, several proteins sustaining an inflammatory reaction were found up-regulated, namely interleukin-1 alpha and beta (IL1A and IL1B), interleukin-36 gamma (IL36G), as well as interleukin enhancer binding factors 2 and 3 (ILF2 and ILF3; Fig. 6c and Supplementary Fig. S7). In line, data bioinformatics processing indicated the regulation of the transcription factor NF-kB in relation to the proteins up-regulated in the cytoplasmic fraction (Fig. 6a).

In the nuclear fraction, the downregulation of squalene synthase FDFT1 was confirmed and accompanied by the decrease of the long-chain-fatty-acid-CoA ligase4 (ACSL4, Fig. 6d). In parallel, downregulation of catenin alpha-1 and beta and of the epidermal growth factor receptor (EGFR) was also observed (Supplementary Fig. S7D). The effect of DON on the lipid synthesis machinery of HEKn cells was evidenced also by the up-regulation of the RNA-binding protein RALY, transcriptional co-factor in cholesterol biosynthesis ((Sallam et al. 2016) Fig. 6d) and by the effect on lipocalin-1 protein (LCN1), which was described in relation to binding/transport of several lipid species (Glasgow and Abduragimovaa 2018) (Supplementary Fig. S7D). Likewise, the Ras-related protein Rab-18, which plays a role in tethering lipid droplets to the endoplasmic reticulum (Dejgaard and Presley 2019), was found up-regulated (Supplementary Fig. S7D). In line with the increase of keratin observed in the cytosolic fraction, the Fos-related antigen 2 (FOSL2) and the transcription factor JUND (binding AP-1 sites) were found up-regulated in the nuclear extract (Wurm et al. 2015) (Fig. 6d).

### Comparison of the biological response triggered by DON in A431 and HEKn cells

**Fig. 7:**
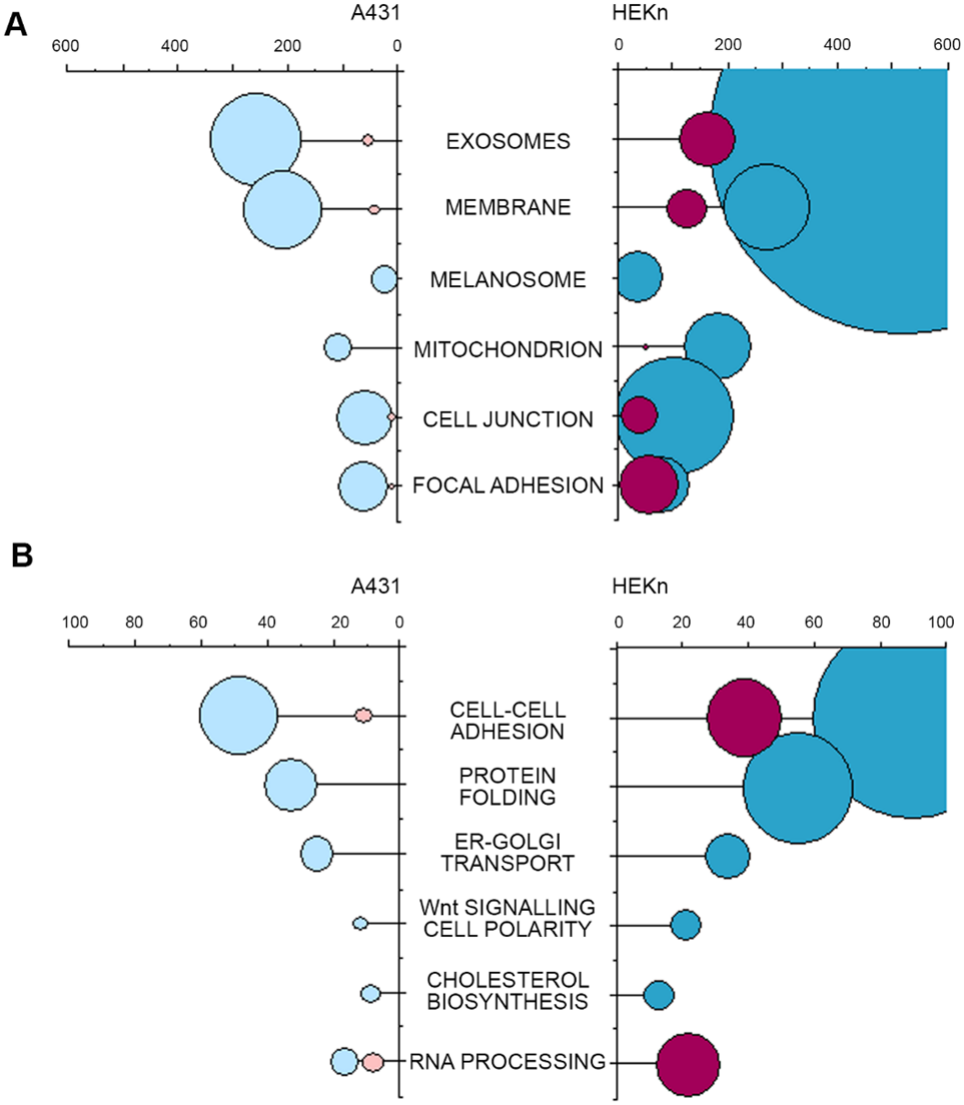
A representation of DAVID functional annotation of cellular components (**a**) and biological processes (**b**) significantly regulated in A431 and HEKn in association with up-regulated proteins (red) and down-regulated proteins (blue). *X* Axes indicate the number of regulated proteins (counts) and the size of the bubbles is equivalent to –Log of the *p* value (FDR Benjamini Hochberg, Supplementary Material Table 4)

Despite the difference in the response typical between tumor and primary cells, our data imply that the lipid synthesis and the related membrane barrier function can be considered as revevant toxicological targets for DON in both skin cell models. Data analysis through DAVID Bioinformatics Resources (Huang da et al. 2009) allowed to clearly highlight the commonalities and the differences between the response of the two cell models in terms of significantly regulated cellular components (Fig. 7a) and biological processes (Fig. 7b). For both A431 and HEKn, significant regulatory events could be traced back to the GO Terms (i) exososomes, (ii) membrane, (iii) cell junctions and (iv) focal adhesions. Interestingly, these cellular components were common denominators for down-regulated proteins in both cell types, but associated with up-regulated proteins predominantly in the primary keratinocytes. Mitochondria, as well as cell-type-specific components such as the melanosomes, were found significantly regulated for both A431 and HEKn, but prevalently associated with the negatively regulated proteins. As for the biological processes, “cell–cell adhesion” was the most affected by the incubation with DON. This was accompanied by the regulation of several pathways connected to membrane homeostasis and cell biomechanical compliance that are essential contributors of skin integrity and barrier function (e.g., (i) cholesterol biosynthesis, (ii) Wnt signaling and (iii) endoplasmic reticulum to golgi transport). In association with the ribosomal-inhibitory potential of DON, protein folding and RNA processing were also found significantly regulated (Fig. 7b).

### Phosphoproteome analysis of DON-treated A431 and HEKn cells

**Fig. 8:**
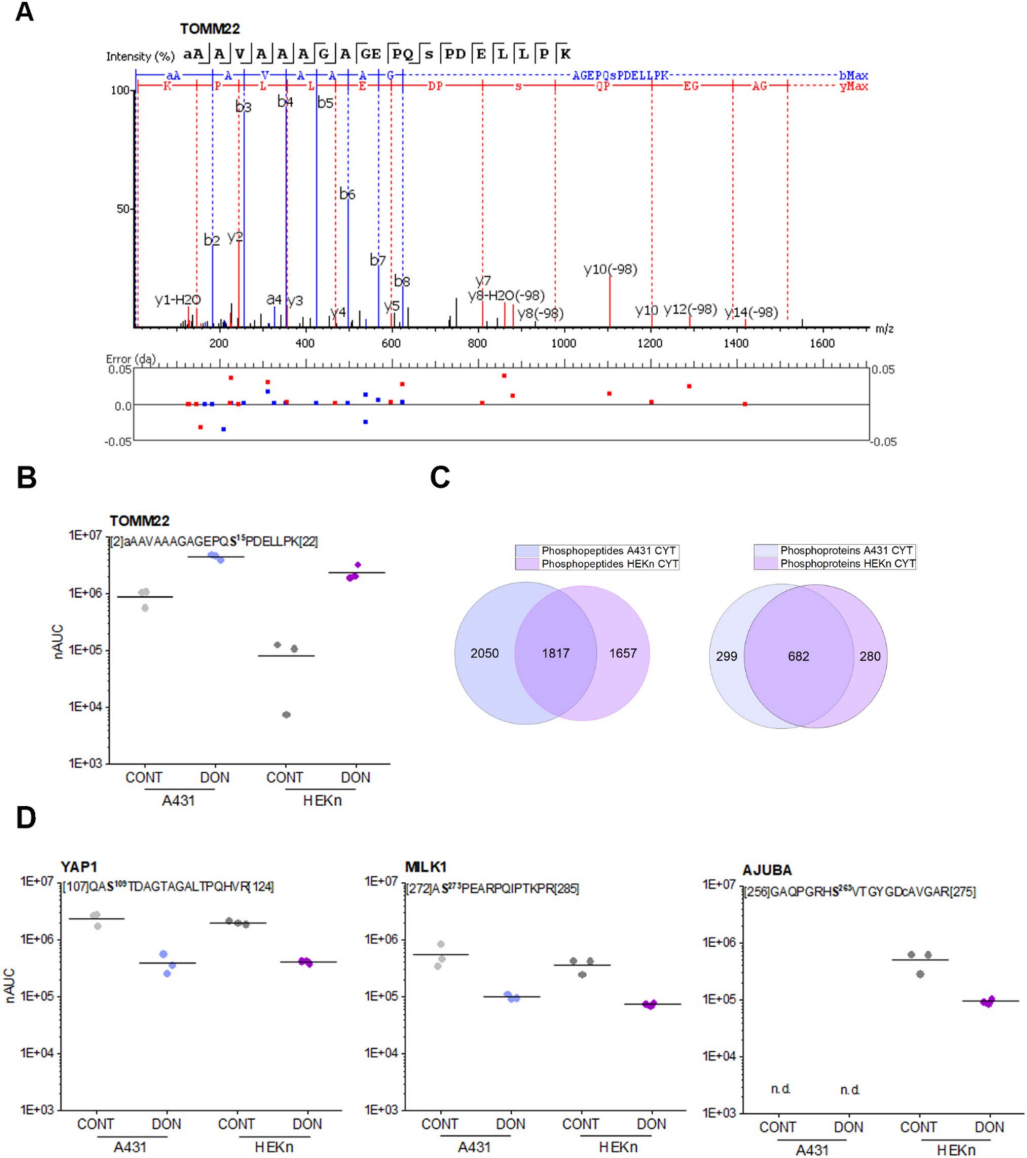
Phosphoproteome analysis based on enrichment via metal oxide affinity chromatography. **a** Interpreted MS2 spectrum of the TOMM22 phosphopeptide with mass deviations observed for all matched fragment ions. **b** DON-induced phosphorylation demonstrated by the indicated phosphopeptide. The position of the peptide sequence within the protein sequence is indicated by numbers in brackets. The phosphorylated amino acid in marked in bold. nAUC, normalized area under the curve. **c** Venn diagrams depicting the number of identified phosphopeptides and phosphoproteins an A431 and HEKn cells, respectively. **d** DON-induced alterations of phosphopeptide abundance values exemplified on phosphopeptides derived from the proteins YAP1, MILK1 and AJUBA

To deepen the understanding of the molecular events downstreaming from the incubation of DON with epidermal cells, we performed also a phosphoproteins analysis based on an affinity enrichment. As a result, out of the cytoplasmic fractions of A431 and nHEK cells, 3867 and 3474 phosphopeptides were identified (10 μM DON, FDR < 0.01; Fig. 8). Of note, one of the most reproducible phosphorylation events involved TOMM22 (Fig. 8a and b). Moreover, the dephosphorylation of YAP1 and MILK1 (MICAL-like protein 1, regulating receptor mediated endocytosis (Abou-Zeid et al. 2011)) was also observed both in A431 and HEKn (Fig. 8d). Intriguigly, the force-sensitive protein AJUBA was found regulated only in HEKn cells (Fig. 8d). Overall, 75/13 phosphopeptides derived from 55/12 phosphoproteins were found significantly regulated in A431/HEKn cells upon DON treatment (Supplementary Material Tables 2–3). Considering A431 cells, most of the significantly regulated phosphoproteins were related to cytoskeletal organization, DNA damage and repair, inflammatory response, lipid binding and membrane organization. 52 of the 75 phosphopeptides were also identified in HEKn cells, with more than 80% of them regulated similarly although without reaching significance. In HEKn cells, most regulatory events were associated with cytoskeletal organization and inflammatory response, in addition to cholesterol biosynthesis, calcium signaling and translation (Supplementary Material Tables 2–3). Here, of the 5 phosphopeptides commonly identified in A431 cells again 80% (4 peptides) were found consistently regulated. Application of a kinase substrate enrichment analysis workflow revealed a positive correlation with mTOR and NEK2 signaling in A431 cells, whereas protein kinase A and C were most prominent in HEKn cells (Supplementary Fig. S8).

## Discussion

DON is one of the most prevalent mycotoxins worldwide, but despite being studied *in depth,* it still offers novel challenges and many aspect of its toxicological profiling are subject of actual research. As for other toxins, proteomic profiling after incubation with DON has been previously performed and it greatly contributed to enhance our understanding of the molecular events sustaining the immunomodulatory function of the toxin (Nogueira da Costa et al. 2011a, b; Pan et al. 2013), as well as its effect at intestinal (Zhang et al. 2016) and hepatic levels (Smith et al. 2018). We have previously demonstrated that DON can have a quite distinctive effect on A431 cells (Del Favero et al. 2018a). It clearly affects, in the cytoplasmic compartment, proteins regulating cell adhesion, cytoskeletal membrane connection and this reflects on the capability of A431 cells to respond to mechanical stimulation (Del Favero et al. 2018a). However, the chain of molecular events leading to this loss of function, as well as the main molecular players connecting the initial ribosomal inhibition to the alteration of cell biophysical properties up to plasma membrane, remained essentially unknown. In this work, we took advantage of untargeted proteomic profiling, including phosphoproteomic analysis, to elucidate intermediate steps connecting the impairment of protein synthesis with molecular functions crucial for skin barrier maintenance.

In line with the known mechanism of action of DON, i.e. ribosomal inhibition (Cundliffe et al. 1974; Ueno 1977), incubation of A431 cells with DON triggered massive deregulation of ribosomal proteins (Fig. 1b); moreover, induction of proteins forming nuclear pore complexes supports an increase of RNA export, possibly as reaction to the ribosomal inhibition triggered by DON (Fig. 1a). Previous analysis of ribosomal-bound proteins and phosphoproteins precipitated after incubation with DON revealed that even incubation times in the order of magnitude of minutes were sufficient to significantly affect translation and protein folding (Pan et al. 2014). Similar findings were described in immune cells (Nogueira da Costa et al. 2011a). In addition, metabolic processes and energy production were also found significantly regulated (Pan et al. 2014). It was recently reported that protein turnover speed is tightly regulated, with proteins forming the mitochondrial respiratory chain being characterized by lower stability/higher turnover in comparison to ribosomal proteins (Zecha et al. 2018). In this light, a massive regulation of mitochondrial proteins upon incubation with DON can be expected (Figs. 1a and 3) and also reflects on the morphology of the organelles (Fig. 4). In addition to the effects on the mitochondrial respiratory chain (Fig. 3a), DON indeed significantly down-regulated at least three crucial proteins of the mitochondrial import machinery, namely TOMM22, TOMM40, TOMM70A (Fig. 3b). TOMM proteins play a central role in the gate system regulating the import of nuclear-encoded proteins into the mitochondrial matrix (Chacinska et al. 2003; Rapaport et al. 1997; Wiedemann et al. 2003). Mitochondrial genes are in fact encoding for a limited number of proteins and the majority needs to be translocated into the organelles (Endo et al. 2003). Overall, alteration of protein synthesis and mitochondrial function retrace the stress signature typical for the unfolded protein response mitochondrial (UPR^mt^). In line with the interpretation that DON could cause mitochondrial stress, phosphorylation of TOMM22 was detected in both A431 and HEKn cells (Fig. 8a, b). This event was previously associated with control of mitophagy (Kravic et al. 2018) and confirms the impact of the toxin on the turnover of the organelles as already observed in other cell types (Bin-Umer et al. 2014; Ren et al. 2020). In line, proteasomal proteins were significantly down-regulated by DON (Fig. 3c, d) as well as other members of the ubiquitination machinery (Fig. 3e). These two components are essential for the cleanup of mistargeted/misfolded proteins (Wrobel et al. 2015) and their reduction upon DON incubation mirrors the cellular stress regarding damaged protein elimination. Of note, proteasomal degradation requires ATP consumption (Finley 2009; Goldberg 2003), thus being dependent on intact mitochondrial function. Hence, it is plausible to hypothesize that DON-induced UPR might result from concomitant (i) inhibition of protein synthesis, (ii) decreased mitochondrial function and ATP production (iii) hampered/overload proteasomal system. Remarkably, UPR^mt^ is associated with increased nuclear translocation of JUN (Callegari and Dennerlein 2018) and DON triggered a concentration dependent increase of the abundance of JUN in the nuclear extract of A431 cells (Fig. 1c). In the complex landscape associated with the UPR^mt^, vast metabolic alteration/adaptations are included (Callegari and Dennerlein 2018; Nargund et al. 2015; Oks et al. 2018). Incubation with DON triggered significant reduction of PDK1 and PDK3 (mitochondrial pyruvate dehydrogenase kinase enzymes 1 and 3), of the fatty aldehyde dehydrogenase (ALDH3A2), as well as a consistent effect on the squalene synthase (FDFT1) and other lipid synthesis-related proteins (Fig. 1, Supplementary Fig. S5). These observations imply an exstensive impairment of the lipid biosynthesis apparatus after exposure to DON and are in good agreement with previous studies describing the capability of DON to impair steroidogenesys (Cortinovis et al. 2014; Guerrero-Netro et al. 2015). In line with the effect on the lipid homeostasis, oPOSSUM engine search of the proteins significantly regulated by the mycotoxin revealed the transcription factor Klf4 as a common denominator of these effects (Fig. 1d, e) and immunofluorescence allowed to describe time and concentration dependent response of the nuclear translocation kinetics (Fig. 2). Klf4 is known to play a crucial role in the maintenance of the barrier function of the skin (Segre et al. 1999) and the validity of this result was extended also to HEKn cells (Fig. 6a, b). Obviously, the membrane plays a major role in defining cellular responses to the extracellular environment (Fuentes and Butler 2012; Lou et al. 2018). Incubation with the mycotoxin significantly altered the morphologic appearance of the cell membrane of A431 cells (Fig. 5b, c), as well as its biophysical properties measured as membrane fluidity (Fig. 5a). Filopodia also belong to membrane structures involved in cell adhesion and migration (Harel and Futerman 1993). Incubation with DON significantly altered the appearance of filopodia size and distribution (Fig. 5). In parallel, the tight junction protein TJP1 (tight junction protein ZO-1, cytosolic fraction 10 µM DON) was found up-regulated, possibly accounting for the areas of cell membrane dye (Cellmask) accumulation visible with confocal microscopy. Indeed, in response to changes of membrane fluidity and decrease of proteins necessary for filopodia formation, it cannot be excluded that other cell–cell adhesion pathways might actually be favored. It was previously described that cells can alternatively tune *N*-cadherin and filopodia, thus enhancing either cell–cell contact or migration (Kroening et al. 2010). Other proteins found regulated in this study seem to confirm this interpretation: for instance, the Ras-related protein Ral-A (RALA; Fig. 3e) important for cell migration (Shi et al. 2006) was found significantly down-regulated in A431 cells upon incubation with DON. A similar behavior could be observed for sorting nexin 9 (SNX9, Fig. 3e) which is involved in the maintenance of cell morphology, membrane/cytoskeleton relation as well as lamellipodia formation (Hicks et al. 2015).

Of note, the effect of DON on the cell membrane could have also been mediated by direct chemical reactivity like in case of lipid peroxidation (Del Favero et al. 2020). So, the definition of the potential involvement of oxidative stress-related pathways was of crucial importance to exclude this possibility. Membrane oxidation and ROS production *per se* can account for changes of membrane biophysical properties (de la Haba et al. 2013), but without the obvious specificity of metabolic involvement. In this respect, H_2_O_2_, included as positive control in the membrane fluidity assay, failed to reproduce the effect mediated by the toxin (Fig. 5a). Indeed, tumor initiating effect of DON on HaCaT cells was reported to be sustained by oxidative stress (Mishra et al. 2016). However, it is known that ROS homeostasis can be altered in tumor cells as result of the malignant transformation (Del Favero et al. 2018b; Toyokuni et al. 1995; Trachootham et al. 2009), this mechanism does not seem to play a major role for A431 cells (Supplementary Fig. S6).

To better highlight the toxicological relevance of our findings also in non-tumor cells, experiments were repeated incubating HEKn primary keratinocytes. Also in this case lipid biosynthesis was influenced by the incubation with DON. For instance, the squalene synthase FDFT1 was consistently down-regulated in nuclear and cytoplasmic compartments (Fig. 6c, d). As a major difference in comparison to A431 cells, the primary keratinocytes presented a marked inflammatory response, with the up-regulation of several cytokines (Fig. 6; Supplementary Fig. S7). This result confirms previous studies on primary keratinocytes (Mishra et al. 2014) and is compatible with the PTM analysis which indicated for HEKn an increased phosphorylation of the substrates of protein kinase A and C (Supplementary Fig. S8). Most importantly, our data are coherent with in vivo studies demonstrating the central role of the inflammatory cascade, and in particular of the protein kinase C, in the dermal toxicity of DON (Mishra et al. 2020b). In line, DON induced in HEKn a significant up-regulation of the nuclear content of Fos-related antigen 2 (FOSL2) as well as of the transcription factor JUND (Fig. 6d). Both components can be traced back to the regulation of the cell redox-inflammatory response (Yin et al. 2017). Moreover, Fra-2/AP-1 interaction is crucial for terminal epidermal differentiation (Wurm et al. 2015) and it is possibly related to the increase in keratine observable in the cytoplasmic fraction (HEKn, Supplementary Fig. S7). In this respect, inhibition of cholesterol synthesis, was previously related to a loss of the cornified envelope of keratinocytes (Ponec et al. 1987) and this mirrors the balance between cornifin A (SPRR1A) and squalene synthase (FDFT1) observed in our experimental conditions (Fig. 6c). Cholesterol homeostasis in keratinocytes is also tightly connected to EGFR (Jans et al. 2004), and we observed significant down-regulation of the protein in HEKn after incubation with DON (Supplementary Fig. S7). Overall, our data pointed toward a substantial dysregulation of cell structural elements (e.g., keratin and cornifin-A) and membrane, possibly tipping the balance toward a more age-prone and “brittle” cell phenotype. This interpretation is sustained also by the morphological charachterization of A431 cells perfomed by confocal microscopy (Figs. 2 and 5). Analysis of the cellular components and biological processes regulated by DON revealed a clear prevalence in the alteration of cellular properties necessary to cope with biomechanical stimulation and respective mechanotransduction (Fig. 7). In line, mechano-sensitive transcription factors like YAP1 were also regulated (Fig. 8b). Dephosphorilation of YAP1 is associated with its activation and nuclear translocation (Totaro et al. 2018) and YAP was already described to cooperate with other transcription factors like AP-1 (up-regulated in both A431 Fig. 1c and HEKn, Fig. 6d) to orchestrate cell motility (Liu et al. 2016). Along this line, AJUBA phosphorylation seems to be crucial to ensure its effect on cell adhesion (Nola et al. 2011). AJUBA was decribed in the regulation of epithelial morphogenesis and response to tensional forces (Razzell et al. 2018) as well as in the integration of pro-inflammatory signaling (Feng and Longmore 2005), thus elegantly connecting mechanisms particularly affected in HEKn after incubation with DON (Fig. 8d).

In conclusion, we delineated a chain of events linking ribosomal inhibition, mitochondrial function, lipid metabolism to membrane structure and biophysical properties in A431 cells. Moreover, we demonstrated that lipid synthesis and cell adhesion are severely impaired by DON also in primary skin HEKn keratinocytes. From the toxicological perspective, these data represent an important insight in the biological effects of DON. In fact, considering DON to be produced by *Fusarium* spp already in the fields, this might relate to potential occupational exposure or health-related effects during harvest and food processing. Moreover, these data open new perspectives in the interpretation of the combinatory effects of DON with other toxins targeting the cell membrane, such as for instance fumonisins (Ferrante et al. 2002; Harel and Futerman 1993; Wang et al. 1991; Yoo et al. 1992), and open new intriguing questions in the evaluation of the effects of *Fusarium* toxins at cellular level.

## Supplementary Information

Below is the link to the electronic supplementary material.

Supplementary file1 (PDF 820 kb)

Supplementary file2 (PDF 1004 kb)

## Data Availability

The mass spectrometry proteomics data comprising cytoplasmic fraction of DON-treated (10 µM) and untreated A431 cells as well as nuclear extracts (all experimental conditions) have been deposited to the ProteomeXchange Consortium via the PRIDE (Vizcaino et al. 2016) partner repository with the dataset identifier PXD011474. The data obtained with HEKn cells (all experimental conditions) were archived with the identifier PXD013613. Data describing the concentration-dependent effect of DON on the cytoplasmic compartment of A431 cells have been deposited previously with identifier PXD008996 and (Del Favero et al. 2018a). All data are available at http://www.proteomexchange.org/.

## Abbreviations

DON: Deoxynivalenol
MEM: Minimum essential medium
PMSF: Phenylmethylsulfonyl fluoride
TE: Tris EDTA
NP-40: Nonidet P-40
IAA: Iodoacetamide
FA: Formic acid
ACN: Acetonitrile
HCD: Higher collisional dissociation
LFQ: Label-free quantification
PPM: Parts per million
FDR: False discovery rate
ROS: Reactive oxygen species
DCF-DA: 2′,7′-Dichlorodihydrofluorescein diacetate
CYT: Cytoplasmic fraction
MOAC: Metal oxide affinity chromatography
NE: Nuclear extracts
UPR: Unfolded protein response
HEKn: Human epidermal keratinocytes, neonatal
