# Supplementary material for: Exploring the dermotoxicity of the mycotoxin deoxynivalenol: combined morphologic and proteomic profiling of human epidermal cells reveals alteration of lipid biosynthesis machinery and membrane structural integrity relevant for skin barrier function
Source: Arch Toxicol. 2021 Apr 23;95(6):2201–21. doi: 10.1007/s00204-021-03042-y (PMC8166681; doi:10.1007/s00204-021-03042-y)
Supplement: Supplementary file 1 — Supplementary file1 (PDF 820 kb) [file 204_2021_3042_MOESM1_ESM.pdf]

Exploring the dermotoxicity of the mycotoxin deoxynivalenol: combined morphologic and proteomic profiling of human epidermal cells reveals alteration of lipid biosynthesis machinery and membrane structural integrity relevant for skin barrier function

Giorgia Del Favero[1,2]*‡, Lukas Janker[3,4]*, Benjamin Neuditschko[3,5], Julia Hohenbichler[1], Endre Kiss[2], Lydia Woelflingseder[1], Christopher Gerner[2,3,4]‡, Doris Marko[1,2]

1 Department of Food Chemistry and Toxicology, Faculty of Chemistry, University of Vienna. Währingerstr. 38-40, 1090 Vienna, Austria

2 Core Facility Multimodal Imaging, Faculty of Chemistry University of Vienna. Währingerstr. 38-40, 1090 Vienna, Austria

3 Department of Analytical Chemistry, Faculty of Chemistry, University of Vienna. Währingerstr. 38-40, 1090 Vienna, Austria

4 Joint Metabolome Facility, Faculty of Chemistry University of Vienna. Währingerstr. 38-40, 1090 Vienna, Austria

5 Department of Inorganic Chemistry, Faculty of Chemistry, University of Vienna. Währingerstr. 42, 1090 Vienna, Austria

*These Authors contributed equally to the work.

‡ Correspondence to GDF giorgia.del.favero@univie.ac.at and CG christopher.gerner@univie.ac.at

Supplementary Materials

Figure S1

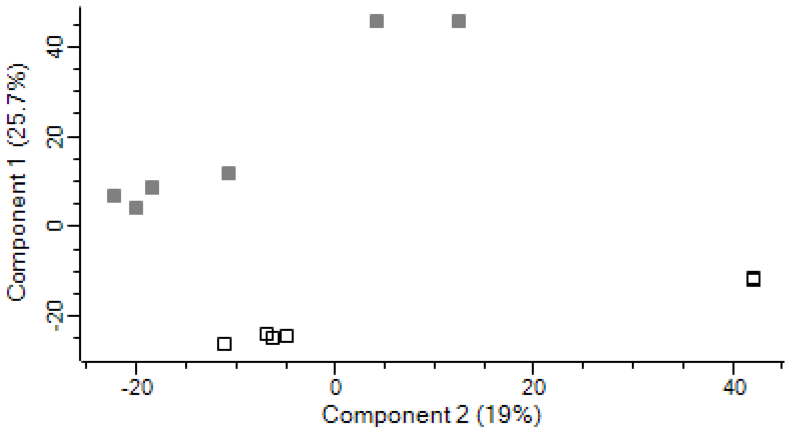

Figure S1. PCA of the cytoplasmic fraction of A431. Solvent controls (white) and 10µM DON (gray).

Figure S2

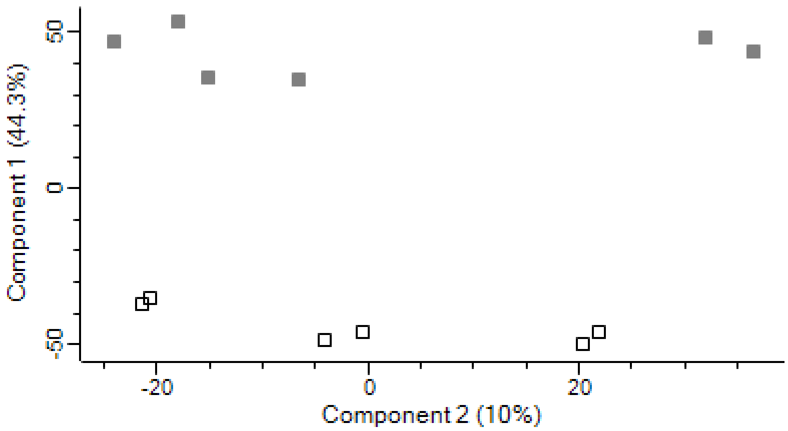

Figure S2. PCA of the nuclear extract of A431. Solvent controls (white) and 10µM DON (gray).

Figure S3

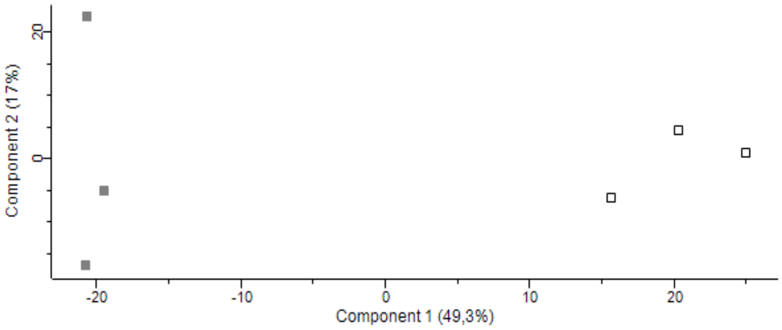

Figure S3. PCA of the cytoplasmic fraction of HEKn. Solvent controls (white) and 10µM DON (gray).

Figure S4

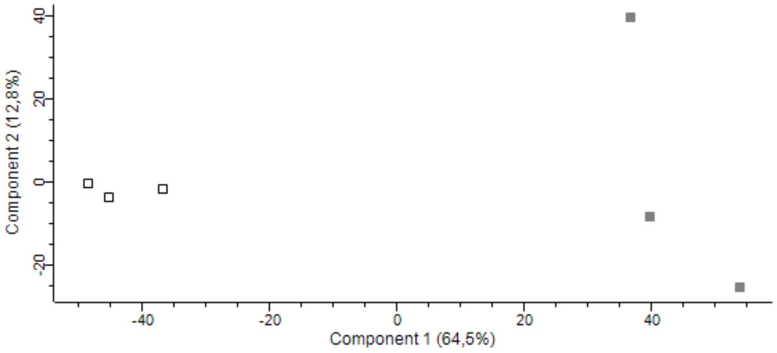

Figure S4. PCA of the nuclear extract of HEKn. Solvent controls (white) and 10µM DON (gray).

Figure S5

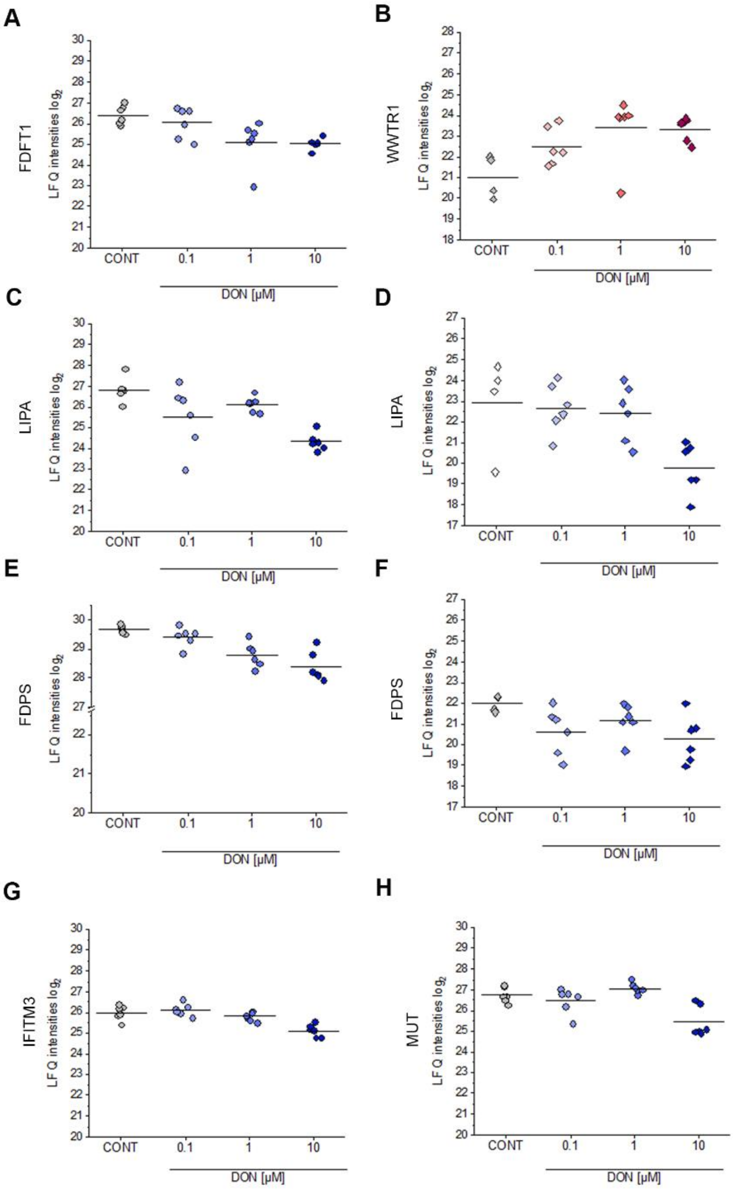

Figure S5. Concentration dependent effect of DON on A431 cells. Concentration dependent effect of the mycotoxin (0.1, 1, 10 µM) on the cytoplasmic fraction (circles) and on the nuclear extract (diamonds) of A431 cells. A. Squalene synthase (FDFT1); B. WW domain-containing transcription regulator protein 1 (WWTR1). C-D Lysosomal acid lipase/cholesteryl ester hydrolase (LIPA); E-F Farnesyl pyrophosphate synthase (FDPS); G. Interferon-induced transmembrane protein 3 (IFITM3). H. Methylmalonyl-CoA mutase (MUT).

Figure S6

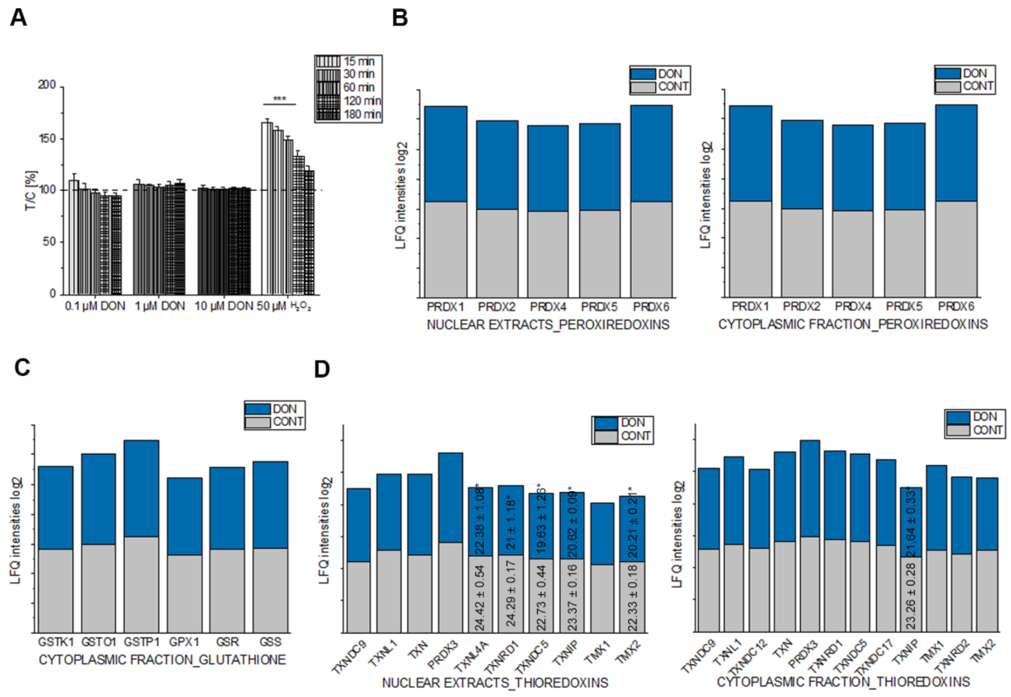

Figure S6. Impact of DON on oxidative stress response in A431 cells. A. Time and concentration dependent effect of DON and H_2O_2 measured by DCF-DA assay. Data are expressed as mean of n > 3 independent experiments performed in technical quadruplicates. B. Effect of DON on peroxiredoxins (nuclear extracts NE; cytoplasmic fraction CYT). C. Effect of DON on glutathione (cytoplasmic fraction CYT). D. Effect of DON on thioredoxins (nuclear extracts NE; cytoplasmic fraction CYT). Significant differences between controls (CONT, grey) and 10 µM DON (DON, blue) are indicated on the respective bars as mean LFQ values ± standard deviation of the 3 independent biological replicates.

Measurement of intracellular reactive oxygen species (ROS)

Time-dependent intracellular reactive oxygen species (ROS) production after exposure to DON (0.1-10 µM) was measured with the DCF-DA assay. A431 cells were incubated with 50 µM of DCF-DA (100 µl, 30 min, 37 °C, Sigma-Aldrich). Afterwards, cells were washed with PBS and exposed to the toxin. Subsequently, measurements were carried out in phenol-red free DMEM (Sigma-Aldrich). Fluorescence was measured with a multi-mode microplate reader Cytation3 Imaging Multi-Mode Reader (ex./em. 480 /520 nm; BioTek, Winooski, VT, USA). DCF-DA data are expressed as the means ± standard error of the mean (S.E.M.) H_2O_2 (50 µM) was included as positive control.

Figure S7

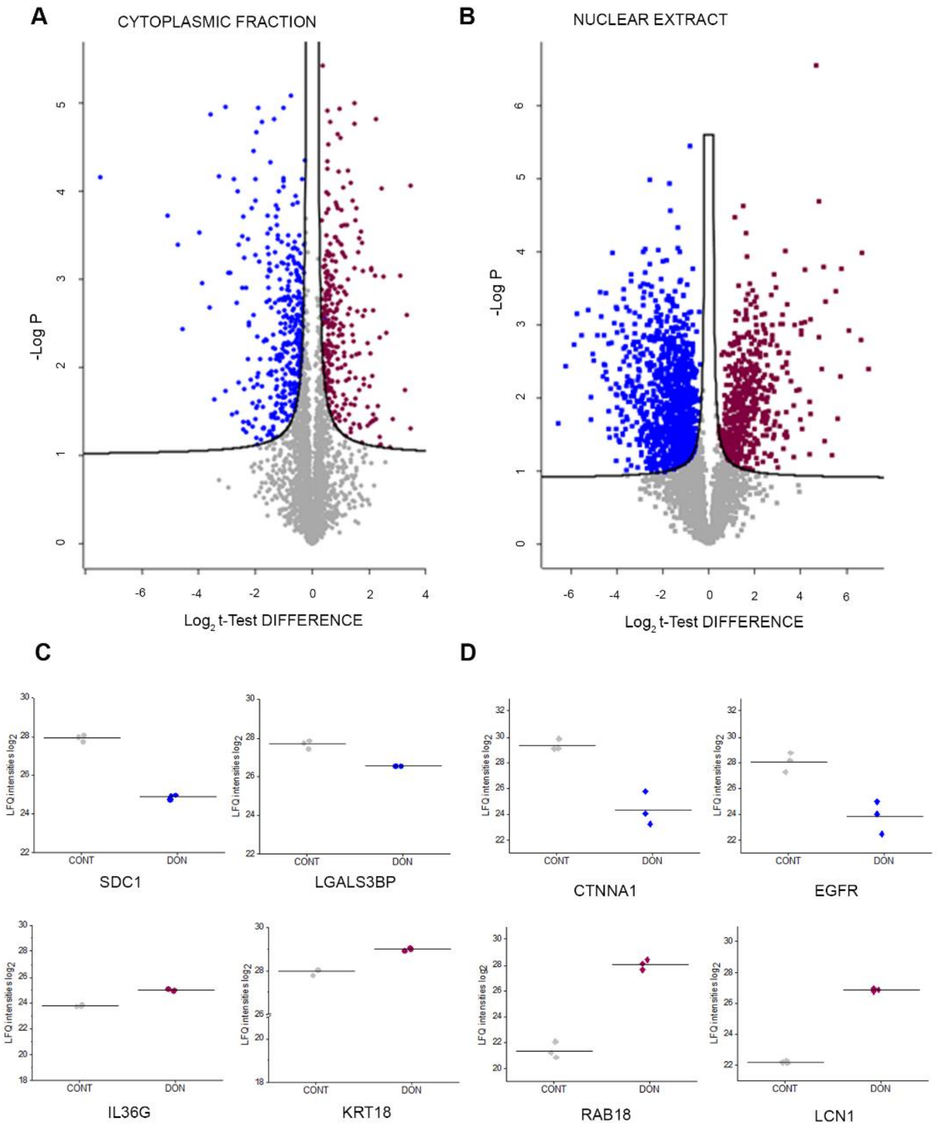

Figure S7. Volcanos plots depicting significant protein regulations between controls (CONT) and 10µM DON in the cytoplasmic fraction of HEKn cells (A) and nuclear extracts (B). C. Selected regulated proteins in the cytoplasmic fraction: syndecan-1 (SDC1), galectin-3-binding protein (LGALS3BP) keratin, type I cytoskeletal 18 (KRT18), interleukin-36 gamma (IL36G). D. Selected regulated proteins in the nuclear extracts: Catenin alpha-1 (CTNNA1), epidermal growth factor receptor (EGFR), ras-related protein Rab-18 (RAB18), lipocalin-1 (LCN1).

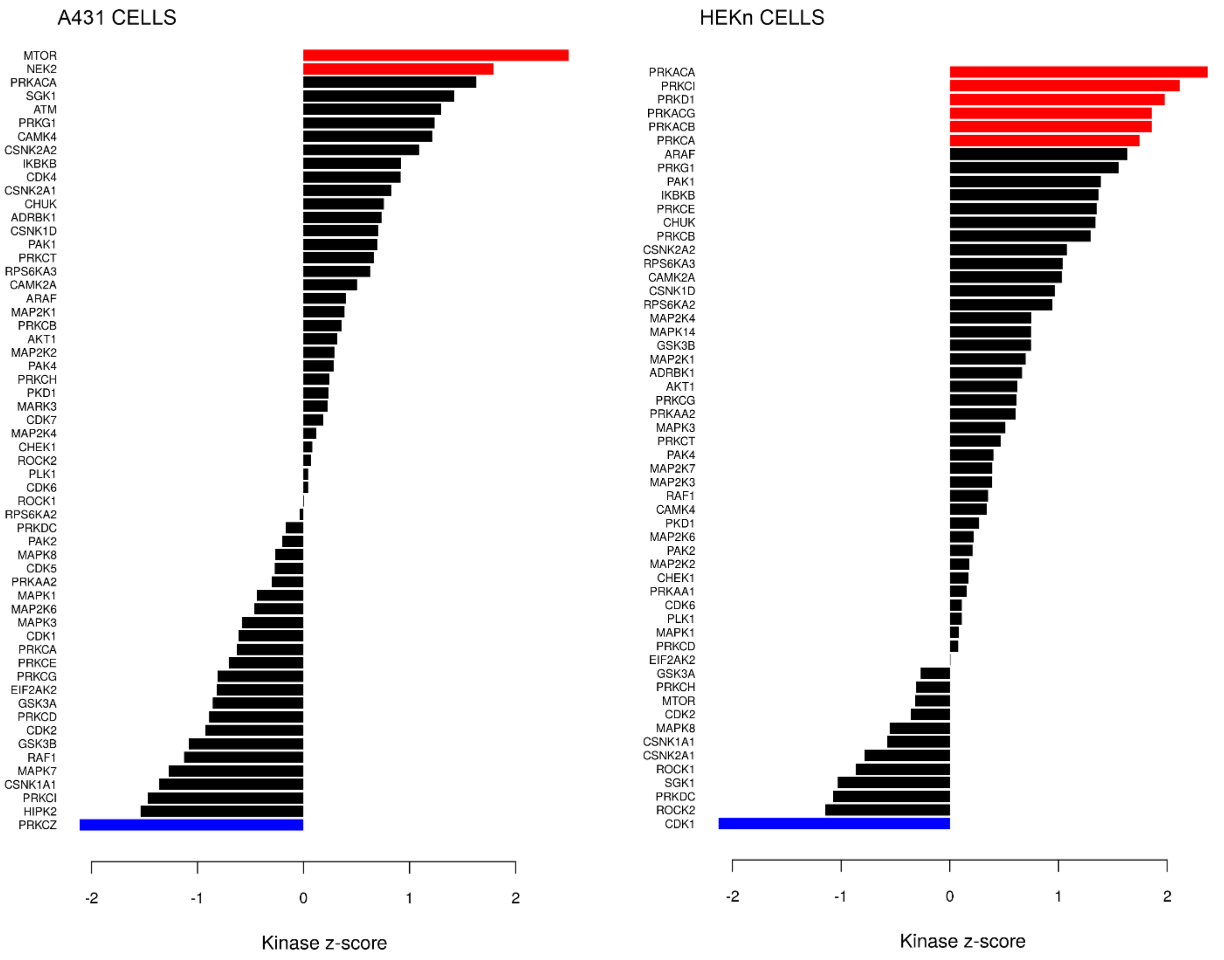

Figure S8. Waterfall plots depicting the association between kinases and phosphorylation events modified by incubation with 10µM DON (24h). Substrate enrichment in comparison to controls is indicated in red, substrate decline is indicated in blue.
