# Supplementary material for: Exploring the dermotoxicity of the mycotoxin deoxynivalenol: combined morphologic and proteomic profiling of human epidermal cells reveals alteration of lipid biosynthesis machinery and membrane structural integrity relevant for skin barrier function
Source: Arch Toxicol. 2021 Apr 23;95(6):2201–21. doi: 10.1007/s00204-021-03042-y (PMC8166681; doi:10.1007/s00204-021-03042-y)
Supplement: Supplementary file 2 — Supplementary file2 (PDF 1004 kb) [file 204_2021_3042_MOESM2_ESM.pdf]

Exploring the dermotoxicity of the mycotoxin deoxynivalenol: combined morphologic and proteomic profiling of human epidermal cells reveals alteration of lipid biosynthesis machinery and membrane structural integrity relevant for skin barrier function

Giorgia Del Favero[1,2]*‡, Lukas Janker[3,4]*, Benjamin Neuditschko[3,5], Julia Hohenbichler[1], Endre Kiss[2], Lydia Woelflingseder[1], Christopher Gerner[2,3,4]‡, Doris Marko[1,2]

1 Department of Food Chemistry and Toxicology, Faculty of Chemistry, University of Vienna. Währingerstr. 38-40, 1090 Vienna, Austria

2 Core Facility Multimodal Imaging, Faculty of Chemistry University of Vienna. Währingerstr. 38-40, 1090 Vienna, Austria

3 Department of Analytical Chemistry, Faculty of Chemistry, University of Vienna. Währingerstr. 38-40, 1090 Vienna, Austria

4 Joint Metabolome Facility, Faculty of Chemistry University of Vienna. Währingerstr. 38-40, 1090 Vienna, Austria

5 Department of Inorganic Chemistry, Faculty of Chemistry, University of Vienna. Währingerstr. 42, 1090 Vienna, Austria

*These Authors contributed equally to the work.

‡ Correspondence to GDF giorgia.del.favero@univie.ac.at and CG christopher.gerner@univie.ac.at

Supplementary Table 1

Raw data bioinformatics oPOSSUM analysis

Transcription factors responsible for regulation of proteins in nuclear extracts of A431 cells

A431_NE_10µM_DON vs A431_NE_CON significantly up-regulated proteins submitted to oPOSSUM

TF	Class	Family	IC	GC Content	Target gene hits	Target TFBS	Z-score
Egr1	Zinc-coordinating	BetaBetaAlpha-zinc finger	14,456	0,739	10	23	7,692
ELK1	Winged Helix-Turn-Helix	Ets	8,812	0,568	19	83	7,597
Klf4	Zinc-coordinating	BetaBetaAlpha-zinc finger	12,618	0,771	21	122	5,789
MIZF	Zinc-coordinating	BetaBetaAlpha-zinc finger	13,197	0,610	5	6	5,626
MYC::MA	Zipper-Type	Helix-Loop-Helix	14,237	0,602	5	9	5,283

A431_NE_10µM_DON vs A431_NE_CON significantly down-regulated proteins submitted to oPOSSUM

TF	Class	Family	IC	GC Content	Target gene hits	Target TFBS	Z-score
Zfx	Zinc-coordinating	BetaBetaAlpha-zinc finger	13,077	0,749	97	340	19,467
Klf4	Zinc-coordinating	BetaBetaAlpha-zinc finger	12,618	0,771	135	837	18,413
GABPA	Winged Helix-Turn-Helix	Ets	13,335	0,647	94	215	13,885
Egr1	Zinc-coordinating	BetaBetaAlpha-zinc finger	14,456	0,739	68	132	12,897
Tcfcp2l1	Other	CP2	11,650	0,609	95	303	12,883

Transcription factors responsible for regulation of proteins in HEKn cells

Nuclear Extracts

HEKn_NE_10µM_DON vs HEKn_NE_CON significantly up-regulated proteins submitted to oPOSSUM

TF	Class	Family	IC	GC Content	Target gene hits	Target TFBS	Z-score
ELK1	Winged Helix-Turn-Helix	Ets	8,812	0,568	372	1569	18,433
Klf4	Zinc-coordinating	BetaBetaAlpha-zinc finger	12,618	0,771	370	2440	16,553
TBP	Beta-sheet	TATA-binding	10,086	0,377	264	954	15,841

HEKn_NE_10µM_DON vs HEKn_NE_CON significantly down-regulated proteins submitted to oPOSSUM

TF	Class	Family	IC	GC Content	Target gene hits	Target TFBS	Z-score
GABPA	Winged Helix-Turn-Helix	Ets	13,335	0,647	555	1248	13,658
Zfx	Zinc-coordinating	BetaBetaAlpha-zinc finger	13,077	0,749	562	1913	13,284
Klf4	Zinc-coordinating	BetaBetaAlpha-zinc finger	12,618	0,771	773	4964	11,79

Cytoplasmic Fraction

HEKn_CYT_10µM_DON vs HEKn_CYT_CON significantly up-regulated proteins submitted to oPOSSUM

TF	Class	Family	IC	GC Content	Target gene hits	Target TFBS	Z-score
Zfx	Zinc-coordinating	BetaBetaAlpha-zinc finger	13,077	0,749	157	491	22,567
Klf4	Zinc-coordinating	BetaBetaAlpha-zinc finger	12,618	0,771	196	1188	18,93
NF-	Ig-fold	Rel	13,345	0,621	130	297	18,289
SP1	Zinc-coordinating	BetaBetaAlpha-zinc finger	11,129	0,82	187	1066	17,674
NFYA	Other Alpha-Helix	NFY CCAAT-binding	12,925	0,523	93	147	15,538

HEKn_CYT_10µM_DON vs HEKn_CYT_CON significantly down-regulated proteins submitted to oPOSSUM

TF	Class	Family	IC	GC Content	Target gene hits	Target TFBS	Z-score
Klf4	Zinc-coordinating	BetaBetaAlpha-zinc finger	12,618	0,771	269	2093	32,891
SP1	Zinc-coordinating	BetaBetaAlpha-zinc finger	11,129	0,82	246	1785	23,131
ZNF354C	Zinc-coordinating	BetaBetaAlpha-zinc finger	8,958	0,615	297	4480	22,447
TP53	Zinc-coordinating	Loop-Sheet-Helix	26,239	0,603	2	2	21,006
MZF1_1-4	Zinc-coordinating	BetaBetaAlpha-zinc finger	8,586	0,725	293	4270	19,449
Tcfcp2l1	Other	CP2	11,65	0,609	204	734	19,425

General settings for oPOSSUM search

JASPAR collection(s):	CORE
Taxonomic supergroup(s):	vertebrates (Homo sapiens)
Minimum profile specificity:	8 bits
Conservation cutoff:	0,40
Matrix score threshold:	85%
Upstream sequence length:	5000
Downstream sequence length:	5000
Results returned:	All results sorted by Z-score

Supplementary Table 2

Regulated Phosphopeptides A431 Cells

Peptide	Protein name	Protein function	phosphorylation site conformity	Accession	Significance	Sample Profile	Control A431	10uM_DON A431	Sample Profile HEKn	Control HEKn	10µM DON HEKn	PTM
LTVENS(+79.97)PKQEAGISEGQGTAGEEEEK	Density-regulated protein	translation	pos	O43583	15,66		3,14E+06	7,35E+05	NIL	nd	nd	Phosphorylation (STY)
QAS(+79.97)TDAGTAGALTPQHVR	Transcriptional coactivator YAP1	DNA damage response and repair	pos	P46937	16,19		4,33E+06	7,25E+05		4,94E+06	9,29E+05	Phosphorylation (STY)
AHS(+79.97)SPASLQLGAVSPGTLTPTGVVSGPAATPTAQHLR	Transcriptional coactivator YAP1	DNA damage response and repair	pos	P46937	17,84		1,22E+07	2,49E+06		7,45E+06	3,06E+06	Phosphorylation (STY)
GGDVFGDTSFLSNHGGS(+79.97)SGSTHRSPR	Cdc42 effector protein 1	cytoskeletal organization	neg	Q00587	25,41		1,21E+06	8,87E+04	NIL	nd	nd	Phosphorylation (STY)
AVAEEDNGSIGEETDSS(+79.97)PGRK	Stromal interaction molecule 1	Ca2+ signalling	pos	Q13586	17,06		8,09E+05	1,45E+05	NIL	nd	nd	Phosphorylation (STY)
A(+42.01)AAVAAAGAGEPQS(+79.97)PDELLPK	Mitochondrial import receptor subunit TOM22 homolog	protein transport	pos	Q9NS69	16,69		2,28E+06	1,11E+07		1,79E+05	5,56E+06	Acetylation (N-term); Phosphorylation (STY)
AGDLLEDS(+79.97)PKRPK	Hepatoma-derived growth factor	transcription, signal transduction	pos	P51858	39,13		2,18E+07	2,21E+05	NIL	nd	nd	Phosphorylation (STY)
S(+79.97)LPTTVPES(+79.97)PNYR	La-related protein 1	translation	pos	Q6PKG0	20,08		1,61E+06	8,10E+06		1,24E+06	1,28E+06	Phosphorylation (STY)
GEAAAERPGEAAVASS(+79.97)PSK	Myristoylated alanine-rich C-kinase substrate	cytoskeletal organization	pos	P29966	31,81		7,12E+06	2,04E+05	NIL	nd	nd	Phosphorylation (STY)
FS(+79.97)PGAPGGSGSQPNQK	Zyxin	inflammatory response	pos	Q15942	17,01		1,08E+06	3,14E+05	NIL	nd	nd	Phosphorylation (STY)
RPTPNDDTLDEGVGLVHSNIATEHIPS(+79.97)PAK	Hydroxymethylglutaryl-CoA synthase, cytoplasmic	cholesterol biosynthesis	pos	Q01581	16,88		1,48E+06	3,39E+05	NIL	nd	nd	Phosphorylation (STY)
LPSS(+79.97)PVYEDAAS(+79.97)FK	Src substrate cortactin	cytoskeletal organization	pos	Q14247	19,09		3,13E+05	6,33E+06	NIL	nd	nd	Phosphorylation (STY)
GVTIPYRPKPSSS(+79.97)PVIFAGGQDR	Poly(rC)-binding protein 2	inflammatory response	pos	Q15366	15,82		3,93E+06	9,60E+05		7,22E+05	2,02E+05	Phosphorylation (STY)

Supplementary Table 3

Regulated Phosphopeptides HEKn Cells

Peptide	Protein name	Protein function	phosphorylation site conformity	Accession	Significance HEKn	Sample Profile HEKn	Control HEKn	10uM_DON HEKn	Sample Profile A431	Control A431	10µM DON A431	PTM
S(+79.97)QEADVQDWEFRK	182 kDa tankyrase-1-binding protein	DNA damage response and repair	pos	Q9C0C2	18,13		2,54E+04	3,71E+05	NIL	nd	nd	Phosphorylation (STY)
SFGTRPLS(+79.97)SGFSPEEAQQQDEEFEK	182 kDa tankyrase-1-binding protein	DNA damage response and repair	pos	Q9C0C2	15,1		1,06E+07	4,77E+06		1,01E+07	1,51E+07	Phosphorylation (STY)
SGPKPFSAPKPQTS(+79.97)PSPK	Adenylyl cyclase-associated protein 1	cytoskeletal organization	pos	Q01518	43,1		1,10E+07	1,95E+06		5,10E+07	1,27E+07	Phosphorylation (STY)
HSS(+79.97)YPAGTEDDEGMGEEPSPFR	Bcl2-associated agonist of cell death	phosphorylation in response to survival signal	pos	Q92934	17,52		1,02E+06	2,86E+06		6,26E+05	6,12E+05	Phosphorylation (STY)
AEGAAT(+79.97)EEEGTPKESEPQAAAEPAEAK	Brain acid soluble protein 1	transcription	pos	P80723	37,61		3,34E+04	2,26E+05	NIL	nd	nd	Phosphorylation (STY)
AVQLMQQVASNGATLPSALSAS(+79.97)K	Brain-specific angiogenesis inhibitor 1-associated protein 2	cytoskeletal organization	pos	Q9UQB8	56,28		1,30E+06	2,31E+05		1,49E+06	1,01E+06	Phosphorylation (STY)
ASASRPQPAPADGADPPPAEEPEARPDGEGS(+79.97)PGK	Breakpoint cluster region protein	cytoskeletal organization	pos	P11274	16,45		1,47E+06	2,45E+05		5,94E+06	3,61E+06	Phosphorylation (STY)
M(+42.01)EVAEPS(+79.97)SPTEEEEEEEEHSAEPRPR	BRISC and BRCA1-A complex member 1	DNA damage response and repair	pos	Q9NWV8	19,06		2,95E+05	8,76E+05		2,77E+05	2,54E+05	Acetylation (N-term); Phosphorylation (STY)
TPEELDDS(+79.97)DFETEDFDVR	Catenin alpha-1	cytoskeletal organization	pos	P35221	17,82		1,36E+07	5,09E+06		1,53E+06	1,14E+06	Phosphorylation (STY)
NSNS(+79.97)YGIPEPAHAYAQPQTTTPLPAVSGSPGAAITPLPSTQNGPVFAK	Crk-like protein	activation of MAPK	neg	P46109	24,88		3,77E+06	1,26E+06	NIL	nd	nd	Phosphorylation (STY)
NSNSYGIPEPAHAY(+79.97)AQPQTTTPLPAVSGSPGAAITPLPSTQNGPVFAK	Crk-like protein	activation of MAPK	pos	P46109	23,12		4,16E+06	1,33E+06	NIL	nd	nd	Phosphorylation (STY)
KPVTVSPTTPTS(+79.97)PTEGEAS	Cytoplasmic dynein 1 light intermediate chain 1	cytoskeletal organization	pos	Q9Y6G9	24,11		1,80E+05	5,46E+05	NIL	nd	nd	Phosphorylation (STY)
DFQDYMEPEEGC(+57.02)QGS(+79.97)PQR	Cytoplasmic dynein 1 light intermediate chain 2	cytoskeletal organization	pos	O43237	20,35		7,22E+05	2,72E+05		6,72E+05	9,21E+05	Carbamidomethylation; Phosphorylation (STY)
TPSNTPSAEADWS(+79.97)PGLELHPDYK	Deoxynucleoside triphosphate triphosphohydrolase SAMHD1	DNA damage response and repair	pos	Q9Y3Z3	17,66		7,31E+05	2,85E+05		5,07E+05	5,57E+05	Phosphorylation (STY)
AS(+79.97)APSPNAQVAC(+57.02)DHC(+57.02)LK	E3 ubiquitin/ISG15 ligase TRIM25	inflammatory response	neg	Q14258	18,13		3,70E+05	7,99E+04		4,26E+05	2,95E+05	Phosphorylation (STY); Carbamidomethylation
ASPS(+79.97)PQPSSQPLQIHR	Echinoderm microtubule-associated protein-like 4	cytoskeletal organization	pos	Q9HC35	24,45		1,43E+06	5,00E+05		4,35E+06	3,36E+06	Phosphorylation (STY)
RADLNQGIGEPQS(+79.97)PSRR	EF-hand domain-[illegible]	regulation of [illegible]	pos	Q96C19	23,94		6,61E+05	1,26E+05		2,49E+07	9,60E+06	Phosphorylation (STY)
RADLNQGIGEPQS(+79.97)PSRR	EF-hand domain-containing protein D2	regulation of inflammation	pos	Q96C19	23,94		6,61E+05	1,26E+05		2,49E+07	9,60E+06	Phosphorylation (STY)
QVS(+79.97)ASELHTSGILGPETLR	Epiplakin	cytoskeletal organization	neg	P58107	41,2		2,80E+06	1,22E+07		1,22E+06	1,99E+06	Phosphorylation (STY)
RQVS(+79.97)ASELHTSGILGPETLR	Epiplakin	cytoskeletal organization	pos	P58107	19,58		4,44E+05	3,67E+06		3,15E+05	2,88E+05	Phosphorylation (STY)
RQVSAS(+79.97)ELHTSGILGPETLR	Epiplakin	cytoskeletal organization	neg	P58107	19,18		1,49E+04	1,58E+05		1,15E+06	2,22E+06	Phosphorylation (STY)
TPADTGFAFPDWAYKPESS(+79.97)PGSR	ETS domain-containing transcription factor ERF	regulation of transcription, [illegible]	pos	P50548	24,24		1,81E+06	5,91E+05	NIL	nd	nd	Phosphorylation (STY)

NKPGPNIES(+79.97)GNEDDDASFK	Eukaryotic translation initiation factor 5B	translation initiation	pos	O60841	21,19		8,28E+06	2,82E+06		8,52E+06	1,32E+07	Phosphorylation (STY)
QS(+79.97)FDDNDSEELEDKDSK	Eukaryotic translation initiation factor 5B	translation initiation	pos	O60841	22,87		6,21E+05	1,54E+05		8,51E+05	1,26E+06	Phosphorylation (STY)
LVS(+79.97)PGSANETSSILVESVTR	Filamin-B	cytoskeletal organization	pos	O75369	17,93		2,02E+05	1,52E+06		1,53E+05	2,76E+05	Phosphorylation (STY)
EEKES(+79.97)EDKPEIEDVGSDEEEEKK	Heat shock protein HSP 90-alpha	protein refolding	pos	P07900	18,19		1,26E+06	4,18E+05		9,29E+06	6,24E+06	Phosphorylation (STY)
EEKESEDKPEIEDVGS(+79.97)DEEEEK	Heat shock protein HSP 90-alpha	protein refolding	pos	P07900	30,81		2,18E+07	6,12E+06		3,23E+07	2,98E+07	Phosphorylation (STY)
E(+42.01)R(+.98)EKEIS(+79.97)DDEAEEEK	Heat shock protein HSP 90-beta	protein refolding	pos	P08238	26,36		8,56E+05	1,99E+05		1,41E+07	6,99E+06	Acetylation (N-term); Deamidation (R); Phosphorylation (STY)
MLPHAPGVQMQAIPEDAIPEES(+79.97)GDEDEDDPDKR	Histone deacetylase 1	chromatin	pos	Q13547	22,59		2,49E+06	8,46E+05		5,66E+06	3,80E+06	Phosphorylation (STY)
NC(+57.02)YS(+79.97)ENEEDSSSIDHLSLNQK	Interleukin-1 alpha	inflammatory response	neg	P01583	18,14		9,78E+04	2,81E+05	NIL	nd	nd	Carbamidomethylation; Phosphorylation (STY)
RLS(+79.97)LSQSITDDDLEAIANDS(+79.97)EEEIIKPR	Interleukin-1 alpha	inflammatory response	pos	P01583	29,45		3,74E+06	1,23E+07	NIL	nd	nd	Phosphorylation (STY)
TLS(+79.97)SSSMDLSR	Kinesin light chain 2	microtubule-based transport	pos	Q9H0B6	16,07		4,97E+04	1,27E+05	NIL	nd	nd	Phosphorylation (STY)
KLS(+79.97)SSDAPAQDTGSSAAAVETDASR	Kinesin-like protein KIF21A	microtubuli-based movement	neg	Q7Z4S6	16,91		4,12E+05	1,76E+05		2,24E+05	1,74E+05	Phosphorylation (STY)
GAQPGRHS(+79.97)VTGYGDC(+57.02)AVGAR	LIM domain-containing protein ajuba	cell migration	pos	Q96IF1	35,08		9,32E+05	1,81E+05	NIL	nd	nd	Phosphorylation (STY); Carbamidomethylation
HS(+79.97)YPPALGSPGALAGAGVGAAGPLER	LIM domain-containing protein ajuba	cell migration	neg	Q96IF1	15,56		1,46E+06	3,95E+05		3,99E+05	3,22E+05	Phosphorylation (STY)
RSS(+79.97)QPSPTAVPASDSPPTK	MAP7 domain-containing protein 1	cytoskeleton organisation	pos	Q3KQU3	15,67		1,06E+07	4,60E+06	NIL	nd	nd	Phosphorylation (STY)
AS(+79.97)PEARPQIPTKPR	MICAL-like protein 1	lipid binding protein	neg	Q8N3F8	30,9		6,89E+05	1,50E+05		8,17E+05	1,89E+05	Phosphorylation (STY)
VEQM(+15.99)PQAS(+79.97)PGLAPR	MICAL-like protein 1	lipid binding protein	pos	Q8N3F8	52,4		1,73E+05	2,03E+03		7,12E+05	1,83E+05	Oxidation (M); Phosphorylation (STY)
S(+79.97)AGNIPLSPLAR	Microtubule-associated serine/threonine-protein kinase 4	cytoskeletal organization	pos	O15021	20,02		5,01E+05	1,62E+05	NIL	nd	nd	Phosphorylation (STY)
A(+42.01)AAVAAAGAGEPQS(+79.97)PDELLPK	Mitochondrial import receptor subunit TOM22 homolog	protein transport	pos	Q9NS69	28,25		1,79E+05	5,56E+06		2,28E+06	1,11E+07	Acetylation (N-term); Phosphorylation (STY)
QSIS(+79.97)FSGLPSGR	mRNA decay activator protein ZFP36	transcription	pos	P26651	17,21		6,24E+03	2,50E+05	NIL	nd	nd	Phosphorylation (STY)
EAPAEGEAAEPGS(+79.97)PTAAEGEAASAASSTSSPK	Myristoylated alanine-rich C-kinase substrate	cytoskeletal organization	pos	P29966	21,48		1,33E+06	3,55E+06		1,22E+06	1,59E+06	Phosphorylation (STY)
K(+42.01)GDR(+.98)S(+79.97)PEPGQTWTR	Neuroblast differentiation-associated protein AHNAK	RNA splicing	pos	Q09666	18,43		2,92E+06	1,10E+06		3,06E+06	1,82E+06	Acetylation (N-term); Deamidation (R); Phosphorylation (STY)
DGGRS(+79.97)SPGGQDEGGFMAQGK	Paxillin	actin-membrane attachment	pos	P49023	25,56		5,49E+05	1,71E+05		7,90E+05	4,32E+05	Phosphorylation (STY)
SAEPSPTVMSTS(+79.97)LGSNLSELDR	Paxillin	actin-membrane attachment	pos	P49023	15,94		5,53E+05	1,30E+06	NIL	nd	nd	Phosphorylation (STY)
TSSVSNPQDSVGS(+79.97)PC(+57.02)SR	Paxillin	actin-membrane attachment	pos	P49023	19,75		5,33E+06	1,41E+06		9,93E+05	8,69E+05	Phosphorylation (STY); Carbamidomethylation
SQATSPGQT(+79.97)NGDSSLEVLATR	PDZ and LIM domain protein 2	cytoskeletal organization	neg	Q96JY6	15,36		1,24E+05	4,47E+05	NIL	nd	nd	Phosphorylation (STY)
IS(+79.97)YKDALDR	Plectin	cytoskeletal organization	neg	Q15149	28,14		1,40E+07	4,54E+07	NIL	nd	nd	Phosphorylation (STY)
QQSHFAMMHGGTGFAGIDS(+79.97)SSPEVK	Poly(rC)-binding protein 1	RNA processing	neg	Q15365	18,13		8,20E+04	5,41E+05	NIL	nd	nd	Phosphorylation (STY)
VMT(+79.97)IPYQPMPASSPVIC(+57.02)AGGQDR	Poly(rC)-binding protein 1	RNA processing	neg	Q15365	24,49		5,12E+05	1,66E+05		9,25E+05	3,89E+05	Phosphorylation (STY); Carbamidomethylation

S(+79.97)LGHPEPLSNGRPQGNSR	Presenilin-1	antiapoptotic, response to DNA damage	pos	P49768	20,5		5,11E+05	1,12E+05	NIL	nd	nd	Phosphorylation (STY)
AVSDS(+79.97)FGPGEWDDR	Protein lifeguard 3	antiapoptotic	pos	Q969X1	16,93		9,82E+04	3,11E+04		5,55E+04	3,72E+04	Phosphorylation (STY)
S(+79.97)S(+79.97)PQLDPLRKSPTMEQAVQTASAHLPAPAAVGR	Protein LSM14 homolog A	inflammatory response	pos	Q8ND56	19,22		6,69E+06	2,24E+06		2,52E+06	1,93E+06	Phosphorylation (STY)
SS(+79.97)PQLDPLRKSPTMEQAVQT(+79.97)ASAHLPAPAAVGR	Protein LSM14 homolog A	inflammatory response	pos	Q8ND56	22,19		6,55E+06	2,36E+06	NIL	nd	nd	Phosphorylation (STY)
SQEPIPDDQKVS(+79.97)DDDKEK	Protein LYRIC	inflammatory response	pos	Q86UE4	24,23		8,92E+05	2,16E+05		4,29E+06	1,04E+06	Phosphorylation (STY)
S(+79.97)RSHT(+79.97)SEGAHLDITPNSGAAGNSAGPK	Protein NDRG1	DNA damage response and repair, membrane organization	pos	Q92597	28,48		1,60E+06	2,56E+05		4,07E+06	2,20E+06	Phosphorylation (STY)
SHT(+79.97)SEGAHLDITPNSGAAGNSAGPK	Protein NDRG1	DNA damage response and repair, membrane organization	pos	Q92597	24,54		2,80E+07	8,56E+06		4,07E+06	2,20E+06	Phosphorylation (STY)
GLLAQGLRPES(+79.97)PPPAGPLLNGAPAGES(+79.97)PQPK	Protein Niban 2	antiapoptotic	pos	Q96TA1	25,48		5,95E+06	1,88E+06		1,35E+06	9,12E+05	Phosphorylation (STY)
YHGHS(+79.97)MSDPGVS(+79.97)YR	Pyruvate dehydrogenase E1 component subunit alpha, somatic form, mitochondrial	glycolysis	pos	P08559	36,51		1,22E+06	2,34E+05		6,84E+05	1,73E+06	Phosphorylation (STY)
DTHEDHDT(+79.97)STENTDESNHDPQFEPIVSLPEQEIK	Ran-specific GTPase-activating protein	intracellular transport	pos	P43487	23,08		2,92E+06	5,04E+05		8,94E+06	5,20E+06	Phosphorylation (STY)
DTHEDHDTS(+79.97)TENTDESNHDPQFEPIVSLPEQEIK	Ran-specific GTPase-activating protein	intracellular transport	neg	P43487	46,25		2,94E+06	4,68E+05		8,94E+06	5,20E+06	Phosphorylation (STY)
M(+42.01)EDLDQS(+79.97)PLVS(+79.97)SSDSPPRPQPAFK	Reticulon-4	membrane organisation	neg	Q9NQC3	15,77		5,45E+05	1,52E+05		5,47E+05	2,16E+05	Acetylation (N-term); Phosphorylation (STY)
M(+42.01)EDLDQS(+79.97)PLVSSSDS(+79.97)PPRPQPAFK	Reticulon-4	membrane organisation	pos	Q9NQC3	16,06		4,61E+05	1,28E+05		6,50E+05	2,67E+05	Acetylation (N-term); Phosphorylation (STY)
SSS(+79.97)PELVTHLK	Rho GTPase-activating protein 1	endosome function	pos	Q07960	32,03		6,11E+04	3,58E+05		1,58E+05	2,19E+05	Phosphorylation (STY)
DGGNPFAEPSELDNPFQDPAVIQHRPS(+79.97)R	Secretory carrier-associated membrane protein 3	protein transport	pos	O14828	26,3		3,21E+06	9,60E+05		1,53E+05	1,15E+05	Phosphorylation (STY)
QVAEQGGDLS(+79.97)PAANR	Serine/threonine-protein kinase 10	cell migration	pos	O94804	15,43		2,18E+06	5,98E+05		5,19E+06	3,00E+06	Phosphorylation (STY)
DQLSQNVHALVS(+79.97)FR	SH2 domain-containing protein 5	cytoskeletal organization	neg	Q6ZV89	19,6		3,55E+04	1,73E+05	NIL	nd	nd	Phosphorylation (STY)
S(+42.01)(+79.97)DQEAKPSTEDLGDK	Small ubiquitin-related modifier 1	DNA damage response and repair	pos	P63165	21,31		7,39E+05	2,58E+05		2,47E+05	1,23E+05	Acetylation (N-term); Phosphorylation (STY)
ILGSAS(+79.97)PEEEQEKPILDRPTR	SUZ domain-containing protein 1	MAPK activating	pos	Q7Z422	28,64		2,08E+05	8,85E+05		3,30E+05	7,16E+05	Phosphorylation (STY)
ETRS(+79.97)SSESLIFSGNQGR	Tensin-4	anti-apoptotic, cell migration	neg	Q8IZW8	27,8		6,76E+05	2,04E+04	NIL	nd	nd	Phosphorylation (STY)
A(+42.01)ASAAAASAAAASAASGS(+79.97)PGPGEGSAGGEK	Transcription intermediary factor 1-beta	DNA damage response and repair	pos	Q13263	22,65		7,21E+06	2,57E+06		8,31E+06	5,21E+06	Acetylation (N-term); Phosphorylation (STY)
AHS(+79.97)SPASLQLGAVSPGTLTPTGVVSGPAATPTAQHLR	Transcriptional coactivator YAP1	DNA damage response and repair	pos	P46937	15,73		7,45E+06	3,06E+06	NIL	nd	nd	Phosphorylation (STY)
QAS(+79.97)TDAGTAGALTPQHVR	Transcriptional coactivator YAP1	DNA damage response and repair		P46937	32,38		4,94E+06	9,29E+05		1,22E+07	2,49E+06	Phosphorylation (STY)
QSS(+79.97)FEIPDDVPLPAGWEMAK	Transcriptional coactivator YAP1	DNA damage response and repair	pos	P46937	27,68		7,20E+05	1,15E+05	NIL	nd	nd	Phosphorylation (STY)
QGEDLAHVQHPT(+79.97)GAGPHAQEEDSQEEEEDEEAASR	Transmembrane protein 51		neg	Q9NW97	18,81		2,57E+06	6,19E+05		5,41E+06	1,62E+06	Phosphorylation (STY)
RDS(+79.97)SESQLASTESDKPTTGR	Uncharacterized protein C18orf25	protein degradation	pos	Q96B23	20,64		1,11E+06	3,10E+05		2,28E+06	1,40E+06	Phosphorylation (STY)

Supplementary Table 4

Raw data Figure 7

	**BIOLOGICAL PROCESSES**												
		**=-log P**	HEKN_DOWN	COUNT	**=-log P**	HEKN_UP	COUNT	**=-log P**	A431_DOWN	COUNT	**=-log P**	A431_UP	COUNT
GO:0098609	CELL-CELL ADHESION	40,10568	7,84E-41	90	15,3279	4,70E-16	39	15,67162	2,13E-16	49	2,974694	1,06E-03	11
GO:0006457	PROTEIN FOLDING	22,32422	4,74E-23	55	0	1	0	10,61439	2,43E-11	33	0	1	0
GO:0006888	ER to GOLGI vesicle mediated transport	8,995679	1,01E-09	34	0	1	0	6,657577	2,20E-07	25	0	1	0
GO:0060071	Wnt SIGNALLING/PLANAR CELL POLARITY	6,088842	8,15E-07	21	0	1	0	2,548214	2,83E-03	12	0	1	0
GO:0006695	CHOLESTEROL biosynthetic process	5,688246	2,05E-06	13	0	1	0	3,614394	2,43E-04	9	0	1	0
GO:0006396	RNA PROCESSING	0	1,00E+00	0	12,46597	3,42E-13	22	5,182435	6,57E-06	17	3,935542	1,16E-04	8
	**CELLULAR COMPONENTS**	**=-log P**	HEKN_DOWN		**=-log P**	HEKN_UP		**=-log P**	A431_DOWN		**=-log P**	A431_UP	
GO:0070062	EXTRACELLULAR EXOSOMES	155,8697	1,35E-156	521	21,86012	1,38E-22	164	36,84771	1,42E-37	260	4,390406	4,07E-05	53
GO:0016020	MEMBRANE	34,50446	3,13E-35	271	15,61798	2,41E-16	127	30,47108	3,38E-31	210	3,856985	1,39E-04	43
GO:0042470	MELANOSOME	19,79317	1,61E-20	38	0	1,00E+00	0	10,54821	2,83E-11	24	0	1,00E+00	0
GO:0005739	MITOCHONDRION	27,09044	8,12E-28	181	1,832683	1,47E-02	51	10,43297	3,69E-11	109	0	1,00E+00	0
GO:0005913	CELL-CELL ADHERENS JUNCTIONS	46,9914	1,02E-47	103	14,87615	1,33E-15	41	21,81531	1,53E-22	61	3,112383	7,72E-04	12
GO:0005925	FOCAL ADHESION	22,15614	6,98E-23	80	23,92812	1,18E-24	57	19,5376	2,90E-20	64	1,5867	2,59E-02	10
